# Identification of multiple substrate binding sites in SLC4 transporters in the outward-facing conformation: Insights into the transport mechanism

**DOI:** 10.1016/j.jbc.2021.100724

**Published:** 2021-04-28

**Authors:** Hristina R. Zhekova, Alexander Pushkin, Gülru Kayık, Liyo Kao, Rustam Azimov, Natalia Abuladze, Debra Kurtz, Mirna Damergi, Sergei Yu Noskov, Ira Kurtz

**Affiliations:** 1 Centre for Molecular Simulation, Department of Biological Sciences, University of Calgary, Calgary, Alberta, Canada; 2 Department of Medicine, Division of Nephrology, David Geffen School of Medicine, University of California, Los Angeles, California, USA; 3 Brain Research Institute, University of California, Los Angeles, California, USA

**Keywords:** transporter, membrane transport, SLC4 proteins, Band 3, NBCe1, substrate binding sites, computer modeling, SILCS, molecular dynamics, electrophysiology, BCECF-AM, 2′,7′-bis(2-carboxyethyl)-5(6)-carboxyfluorescein tetrakis(acetoxymethyl) ester, CHARMM, Chemistry at Harvard Macromolecular Mechanics, cryo-EM, cryo-electron microscopy, DIDS, 4,4′-diisothiocyanatostilbene-2, 2′-disulfonic acid, ECL, Enhanced Chemiluminescent, EIPA, 5-(N-ethyl-N-isopropyl)-amiloride, GCMC, Grand Canonical Monte Carlo, hAE1, Human Anion Exchanger 1, HEK293, Human Embryonic Kidney Cell Line 293, hNBCe1, Human Electrogenic Sodium Bicarbonate Cotransporter 1, MD, Molecular Dynamics, NAMD, Nanoscale Molecular Dynamics, NPT, MD ensemble with constant number of particles (N), pressure (P), and temperature (T), NVT, MD ensemble with constant number of particles (N), volume (V), and temperature (T), OF, Outward-Facing, pH_i_, Internal pH, POPC, 1-Palmitoyl-2-oleoylphosphatidylcholine, pRTA, Proximal Renal Tubilar Acidosis, SILCS, Site Identification by Ligand Competitive Saturation, SLC4, Solute Carrier Family 4, TBST, Tris buffered saline, TBSTM, Tris buffered saline with polyethylene glycol sorbitan monolaurate, TM, Transmembrane Segment, VMD, Visual Molecular Dynamics

## Abstract

Solute carrier family 4 (SLC4) transporters mediate the transmembrane transport of HCO_3_^−^, CO_3_^2−^, and Cl^−^ necessary for pH regulation, transepithelial H^+^/base transport, and ion homeostasis. Substrate transport with varying stoichiometry and specificity is achieved through an exchange mechanism and/or through coupling of the uptake of anionic substrates to typically co-transported Na^+^. Recently solved outward-facing structures of two SLC4 members (human anion exchanger 1 [hAE1] and human electrogenic sodium bicarbonate cotransporter 1 [hNBCe1]) with different transport modes (Cl^−^/HCO_3_^−^ exchange *versus* Na^+^-CO_3_^2−^ symport) revealed highly conserved three-dimensional organization of their transmembrane domains. However, the exact location of the ion binding sites and their protein–ion coordination motifs are still unclear. In the present work, we combined site identification by ligand competitive saturation mapping and extensive molecular dynamics sampling with functional mutagenesis studies which led to the identification of two substrate binding sites (entry and central) in the outward-facing states of hAE1 and hNBCe1. Mutation of residues in the identified binding sites led to impaired transport in both proteins. We also showed that R730 in hAE1 is crucial for anion binding in both entry and central sites, whereas in hNBCe1, a Na^+^ acts as an anchor for CO_3_^2−^ binding to the central site. Additionally, protonation of the central acidic residues (E681 in hAE1 and D754 in hNBCe1) alters the ion dynamics in the permeation cavity and may contribute to the transport mode differences in SLC4 proteins. These results provide a basis for understanding the functional differences between hAE1 and hNBCe1 and may facilitate potential drug development for diseases such as proximal and distal renal tubular acidosis.

The solute carrier family 4 (SLC4) transporters mediate the transport of HCO_3_^−^, CO_3_^2−^, Cl^−^, Na^+^, K^+^, H^+^, and NH_3_ + H^+^ across cell membranes and are involved in regulation of important physiological processes such as ion homeostasis, pH balance, and blood pressure (1, 2, 3, 4, 5, 6). The anion exchanger 1 (AE1, SLC4A1) is expressed in the plasma membranes of the erythrocytes and the α-intercalated cells in the collecting duct of nephrons where it mediates Cl^−^/HCO_3_^−^ exchange necessary for carbon dioxide removal in the lungs and systemic acid–base homeostasis (7, 8). In line with other secondary transporters, the human anion exchanger 1 (hAE1) is bidirectional and exhibits various anion/anion transport properties (including self-exchange such as Cl^−^/Cl^−^), depending on the external physiological conditions (9). The AE1 turnover rate of ~50,000 s^−1^ makes it one of the fastest secondary transporters known to date (10), although the mechanism of this high rate is unknown. Another member of this family, SLC4A4, which encodes the human electrogenic sodium bicarbonate cotransporter 1 (hNBCe1), is a sodium coupled transporter involved in electrogenic Na^+^-CO_3_^2−^ symport, necessary for carbonate/bicarbonate absorption in the renal proximal tubule (1, 2, 8). hAE1 and hNBCe1 have a high sequence similarity especially in their transmembrane domain areas (~65% similar residues for transmembrane domains) yet differ significantly in their transport properties (exchanger *versus* symporter). Mutations in hAE1 cause distal renal tubular acidosis and red cell morphological abnormalities whereas mutations in hNBCe1 result in proximal renal tubular acidosis (pRTA) (1, 2, 7, 8). Despite their physiological significance and the large amount of functional mutagenesis data available for these two proteins, their transport mechanisms are poorly understood hindering the studies of disease states at the molecular level and the development of potential pharmacological strategies for treatment of these diseases.

Recently, outward-facing (OF) structures of hAE1 and hNBCe1 were resolved with X-ray diffraction (11) and cryo-electron microscopy (cryo-EM) (12) to 3.5 and 3.9 Å, respectively, and putative substrate binding regions were identified in these structures based on the available functional mutagenesis data (7, 11, 12, 13, 14) and comparison with putative binding sites of several proteins, which feature the same 7 + 7 transmembrane segment (TM) inverted repeat fold of their transmembrane domains (the bacterial uracil transporter, UraA (15), the fungal UapA purine-H^+^ symporter (16), the bacterial H^+^-coupled fumarate symporter SLC26Dg (17), and the plant boron transporter Bor1 (18)). The identity of the specific protein residues involved in substrate coordination in the binding pockets of hAE1 and hNBCe1, however, remains elusive. Moreover, hAE1 and hNBCe1 feature two negatively charged residues (E535 and E681 in hAE1 and D555 and D754 in hNBCe1) in their presumed anion binding pockets, which raises questions about the role of these residues in the control of ion dynamics in the SLC4 protein family, where all members have glutamate or aspartate at these locations (1). The position and orientation of the anion substrates in the proposed binding pockets, which is necessary as a first step toward elucidation of the transport mechanism of the SLC4 family, is still unknown because the available crystal and cryo-EM structures did not resolve bound ions. To assess further the substrate binding motifs in the SLC4 protein family, we performed site identification by ligand competitive saturation (SILCS) (19) mapping of the binding pockets of hAE1 and hNBCe1, followed by a number of exploratory all-atom molecular dynamics (MD) simulations. Our simulations identify two putative substrate binding sites in the large permeation cavity of hAE1 and hNBCe1 and provide novel insights for specific protein–substrate interactions that may explain differences in transport mode between these two proteins. The results from the MD simulations were supported by functional mutagenesis and transport data on 18 hAE1 mutants and 19 hNBCe1 mutants.

## Results

### Identification of areas of high cation and anion affinity in the of state of hAE1 and hNBCe1 from SILCS calculations

**Figure 1:**
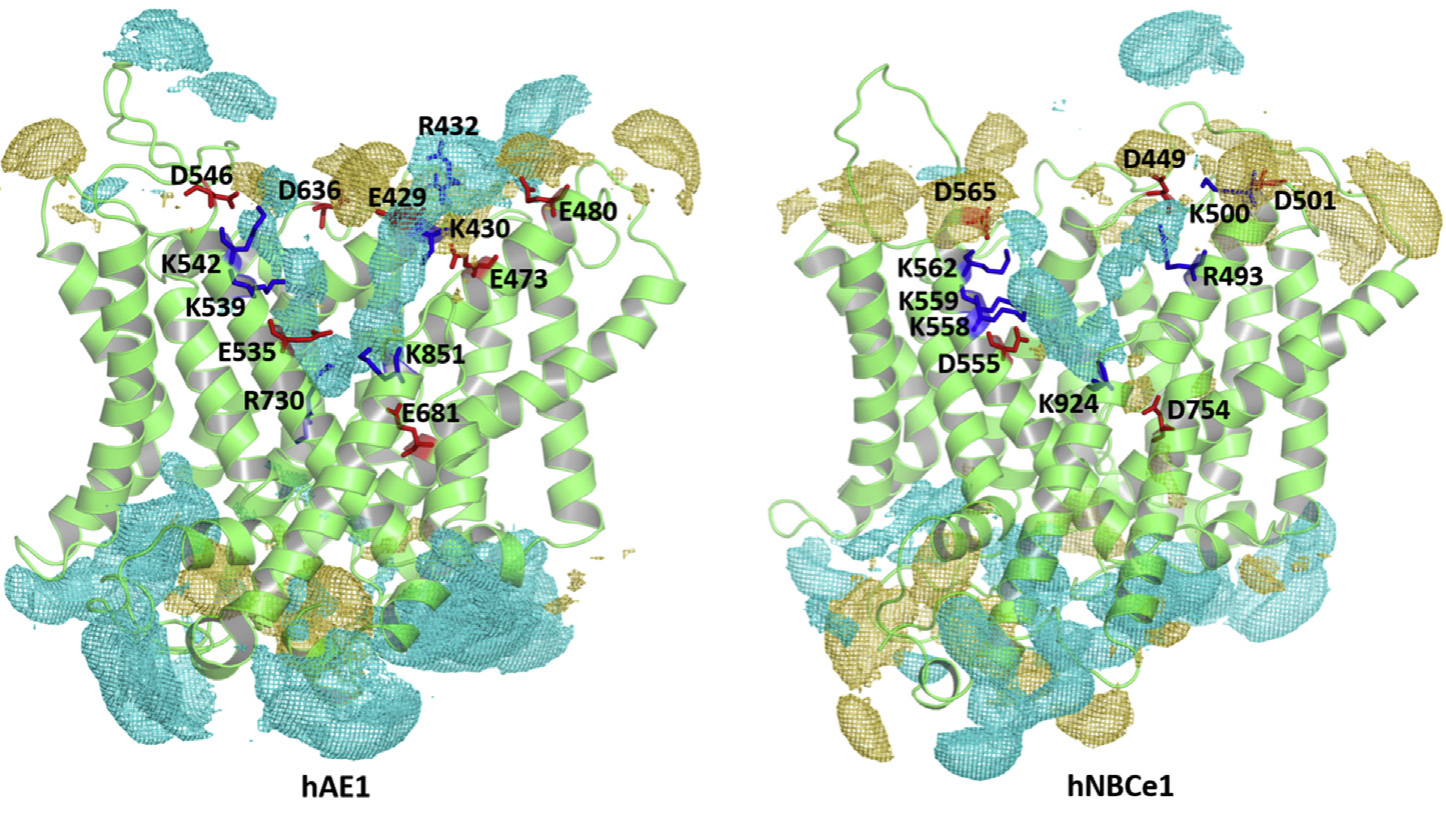
**Ion accessibility in hAE1 and hNBCe1.** SILCS excess density maps (GFE level = −0.7 kcal/mol) of acetyl oxygen (*cyan mesh*) and methylammonium nitrogen (*yellow-orange mesh*) of hAE1 and hNBCe1. The positively and negatively charged residues lining the OF permeation cavity of the two proteins are shown as *blue and red sticks*, respectively. hAE1, human anion exchanger 1; hNBCe1, human electrogenic sodium bicarbonate cotransporter 1; OF, outward-facing.

SILCS calculations employ Grand Canonical Monte Carlo (GCMC) and MD simulations to map protein regions with high affinity for various small solutes (fragments) with different structure and chemical properties, which are present in high concentrations in the simulation solutions. The flooding of the protein with the fragments and the corresponding fragment density maps (FragMaps) produced by SILCS can reveal previously unknown binding pockets and allosteric sites within the 3D protein matrix (19). The SILCS FragMaps approach has been recently employed for identification of binding pockets, including drug-targetable sites in various proteins and for the development of novel drug molecules (20, 21, 22, 23, 24). Figure 1 presents the SILCS fragment maps calculated in hAE1 and hNBCe1 for the oxygen atoms of the acetate anions used as surrogate ions for the SLC4 anionic substrates Cl^−^, HCO_3_^−^, and CO_3_^2−^ (cyan isomesh) and the nitrogen atoms of the methylammonium cations used as surrogate ions for the cotransported Na^+^ (yellow-orange isomesh). Although the acetate and methylammonium ions are larger than the anions normally transported by the SLC4 family proteins, they represent molecular probes with negative and positive charges with already developed parameters as part of SILCS. An enhanced density for these fragments in the protein center indicates that this region could accommodate as well the physiological ions, which are smaller and easier to fit in the putative binding sites. Both hAE1 and hNBCe1 feature a wide well-hydrated OF permeation cavity, lined with several positively charged (blue sticks) and negatively charged (red sticks) residues. These residues control the access of cationic and anionic fragments in the permeation cavity. Acetate can freely traverse the cavity to the protein center in both proteins and presents with a high density in the area of residues K539, R730, and K851 in hAE1 and residues K558, K559, and K924 in hNBCe1 (Fig. 1). Permeation of methylammonium to the protein center occurs to a significantly lower extent in both proteins because of the screening effects of the positively charged lysine and arginine residues in the cavity. However, a sizeable methylammonium density can be discerned in the vicinity of D754 of hNBCe1 implying the existence of a putative Na^+^ binding site in this area. In hAE1, the central R730 residue prevents methylammonium from reaching the protein center, and analogous methylammonium density is not observed in the area of E681.

### Identification of two binding sites, S1 and S2, from 1.2 μs MD simulations of apo-hAE1

**Figure 2:**
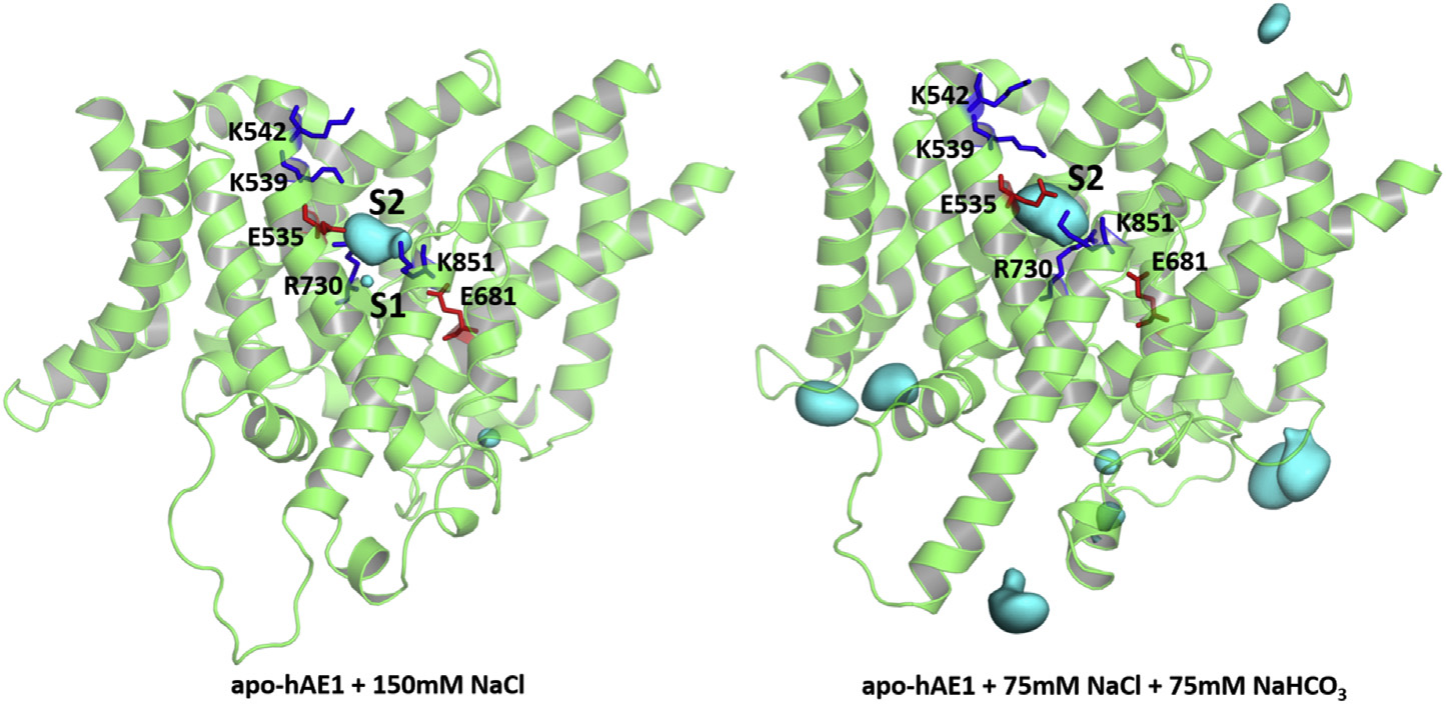
**Binding sites in the OF cavity of hAE1 and hNBCe1.** Anion density maps (isovalue 0.1), identifying the location of two putative binding sites (S1 and S2) in the OF permeation cavity of hAE1, computed from 1.2 μs MD trajectories of apo-hAE1 in 150 mM NaCl solution (Cl^−^ density map) and apo-hAE1 in equimolar 75 mM NaCl + 75 mM NaHCO_3_ solution (overlayed Cl^−^ and HCO_3_^−^ density maps). hAE1, human anion exchanger 1; hNBCe1, human electrogenic sodium bicarbonate cotransporter 1; MD, molecular dynamics; OF, outward-facing.

The acetate and methylammonium SILCS maps in Figure 1 identify areas in the binding pockets of hAE1 and hNBCe1 which could be potentially attractive to the physiological ions transported by these proteins (Cl^−^, CO_3_^2−^, HCO_3_^−^, and Na^+^). To explore further the dynamics of Na^+^, Cl^−^, and HCO_3_^−^ ions in the OF permeation cavity of hAE1 in conditions closer to the physiological environment, we performed two 1.2 μs long all-atom MD simulations starting from an empty (apo-) hAE1 protein embedded in a lipid bilayer solvated in either 150 mM NaCl, approximating the 0.9% NaCl in physiological saline, or an equimolar solution of 75 mM NaHCO_3_ + 75 mM NaCl, with elevated HCO_3_^−^ concentration relative to Cl^−^ (*i.e.*, 1:1 instead of ~1:4 Cl^−^/HCO_3_^−^ ratio (25)) for faster sampling of potentially rare HCO_3_^−^ entry events. During these microsecond-long MD trajectories, HCO_3_^−^ and Cl^−^ ions from the solution penetrate freely and frequently (more than a hundred unique anion entry events, Fig. S1) into the wide well hydrated OF cavity of hAE1, which is consistent with the open nonoccluded conformations of the proteins. Two putative anion binding sites, central (S1) and entry (S2), identified as areas of high anion density (Fig. 2) can be seen in the hAE1 cavity within the area of high acetate oxygen density from our SILCS calculations (Fig. 1). The density at site S1 is significantly smaller which shows that occupation of this site occurs more rarely and for shorter times than site S2. Figure S1 illustrates the frequency of entry and residence times for the HCO_3_^−^ and Cl^−^ ions entering the cavity and central areas of hAE1, which is the location of sites S1 and S2 (Fig. S2), flanked by residues K542, K539, R730, and K851 during the 1.2 μs MD simulations. The portion of MD trajectory steps (in %) during which zero to 3 Cl^−^, HCO_3_^−^ or Na^+^ ions are found in the areas of the permeation cavity of hAE1 and the protein center (location of site S1 and S2) are displayed in Figure S3. The anions often exchange with one another and HCO_3_^−^ routinely persists for more than 100 ns in the protein center (average residence time 151.17 ns), while the presence of Cl^−^ in this area is more short-lived (average residence times 3.96 ns and 24.44 ns in the presence and absence of HCO_3_^−^, respectively, Fig. S1). The long HCO_3_^−^ residence times in the wide well-hydrated protein cavity indicate strong coordination of HCO_3_^−^ in site S2. The cavity of hAE1 accommodates most often one or two Cl^−^ or HCO_3_^−^ anions, and the larger size of HCO_3_^−^ does not hinder its entry within the cavity (Fig. S3). However, the protein central area is occupied by a single anion in most simulation steps in which anions managed to penetrate the cavity to the protein center (with anions mostly residing in the area of site S2 in such cases, Fig. 2). Entry of Na^+^ ions within the cavity occurs in less than 10% of the MD steps. Na^+^ ions did not reach the protein center in the 1.2 μs span of our MD simulations consistent with the results of our SILCS simulations where no significant methylammonium nitrogen density was detected in the protein center of hAE1 (Fig. 1, left). Similar permeation trends for the anions and Na^+^ were observed also in all 250 ns long MD simulations of hAE1 (Table 1 and Fig. S4).

**Table 1:** Cavity residence times of Na^+^ and anion substrate (in ns) in the tested hAE1 and hNBCe1 systems during the 250 ns long MD trajectories

System	Bound ions	Mutation/protonation	Anion binding, ns	Na^+^ binding, ns
hAE1	+HCO_3_^−^ (Trial 1)	-	250	-
	+HCO_3_^−^ (Trial 2)	-	250	-
	+Cl^−^ (Trial 1)	-	250	-
	+Cl^−^ (Trial 2)	-	30^a^	-
	+Na^+^+CO_3_^2−^	-	250	250
	+CO_3_^2−^	-	250	-
	+Na^+^	-	-	0.5
	+Na^+^+Cl^−^	-	78.5^a^	14
	+Na^+^+HCO_3_^−^	-	238	3.5
	+HCO_3_^−^	protE681	43	-
	+Cl^−^	protE681	54.5^a^	-
hNBCe1	+Na^+^+CO_3_^2−^	-	250	250
	+HCO_3_^−^	-	72.5	-
	+Cl^−^	-	7^a^	-
	+CO_3_^2−^	-	3	-
	+Na^+^	-	-	40
	+Na^+^+Cl^−^	-	9^a^	21.5
	+Na^+^+HCO_3_^−^	-	40	35
	+Na^+^+CO_3_^2−^	protD754	250	46.5

hAE1, human anion exchanger 1; hNBCe1, human electrogenic sodium bicarbonate cotransporter 1; MD, molecular dynamics; OF, outward-facing.

a Indicates the cavity residence time of the Cl^−^ ion, bound to the protein at the very beginning of the MD simulation. This Cl^−^ ion is eventually replaced by another Cl^−^ from the surrounding solution. The Cl^−^ ions from the solution permeate freely the large hydrated OF cavities of hAE1 and hNBCe1, and there are multiple binding/unbinding events involving different Cl^−^ ions (see Fig. S4 for more details). Despite their mobility, a Cl^−^ ion is present at site S1 or S2 for the majority of the MD steps in the hAE1 systems leading to a pronounced anion density at these sites.

### MD simulations for assessment of protein–ion binding motifs

**Figure 3:**
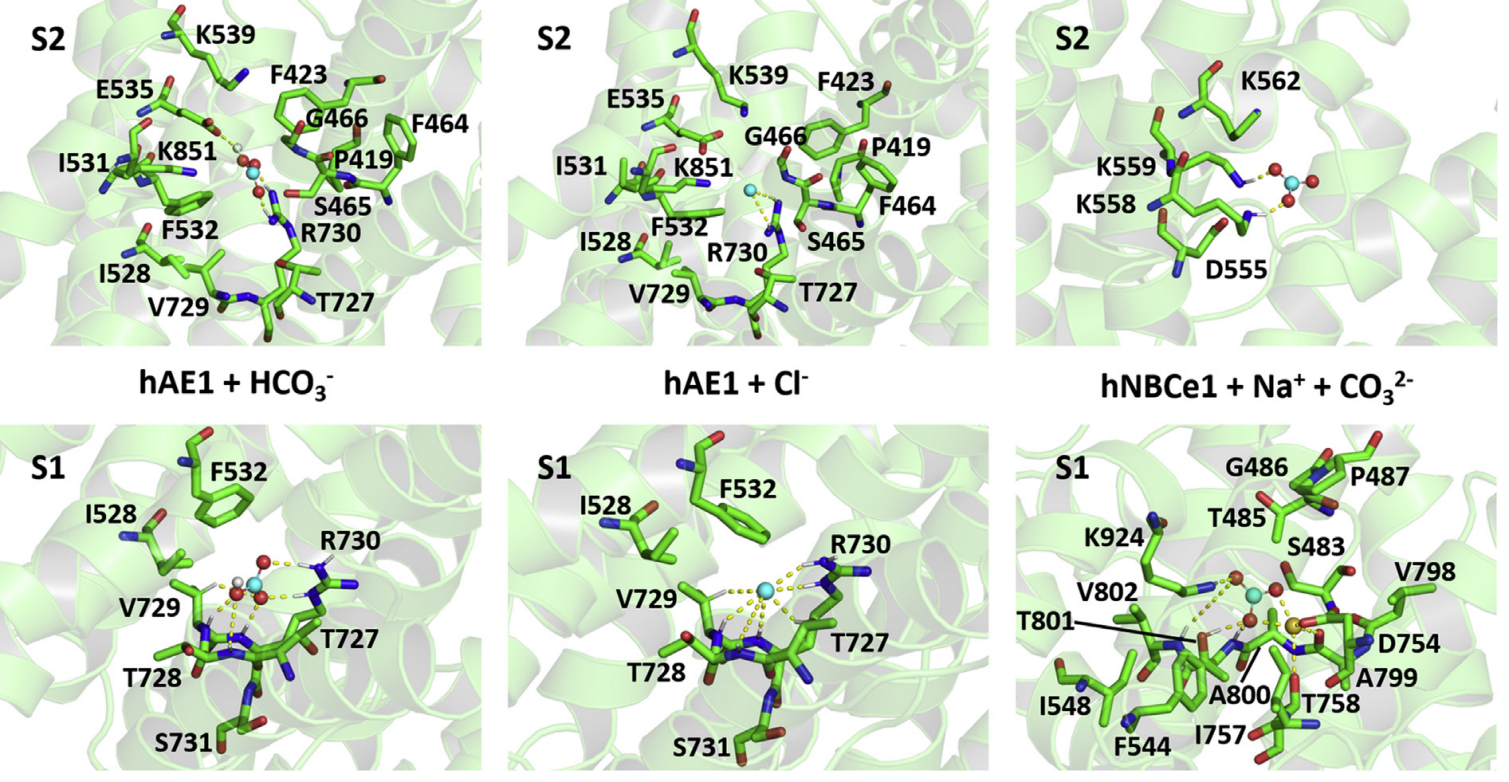
**Sites S1 and S2 in hAE1 and hNBCe1.** Coordination of HCO_3_^−^, Cl^−^, CO_3_^2−^, and Na^+^ ions in sites S1 and S2 in hAE1 and hNBCe1 identified from ion density maps (Figs. 1 and 2) and contact frequency analysis (Fig. S7). The distances between the ions and their closest coordinating residues are indicated with yellow dashed lines. hAE1, human anion exchanger 1; hNBCe1, human electrogenic sodium bicarbonate cotransporter 1.

Considering the position of the two anion binding sites identified within the protein cavity from SILCS maps of hAE1 and hNBCe1 and 1.2 μs MD simulations on the apo-hAE1 system, it is likely that the entry site S2 is a transient binding site, serving as an attractor of anions from the extracellular solution, whereas the central site S1 provides the required substrate binding for the initiation of an ion translocation event. To assess in more detail the amino acid residues of the central site S1 and to probe the behavior of different ions bound to it, we conducted a number of 250 ns MD simulations starting from hAE1 or hNBCe1 loaded with different combinations of physiological ions. The ion/substrate binding stoichiometries studied in the current work include a single anion (CO_3_^2−^, HCO_3_^−^, or Cl^−^), a single cation (Na^+^), and different 1:1 cation:anion combinations (Table 1) in accordance with previous functional data (7, 26, 27). Because the solutions used in our simulations contain Na^+^ and Cl^−^, other binding stoichiometries resulting from simultaneous entry of more than one cation or anion into the cavity of the studied systems could potentially be observed during the course of our MD trajectories. The initial anion placement in the protein center was guided by hAE1 and hNBCe1 SILCS maps for acetate oxygen and methylammonium nitrogen (Fig. 1), the anion density identifying site S1 in the 1.2 μs MD simulations of apo-hAE1 with 150 mM NaCl (Fig. 2), and Poisson-Boltzmann electrostatic maps (Fig. S5) of the binding pockets of hAE1 and hNBCe1 (see Experimental procedures). All systems were then subjected to 250 ns long unconstrained all-atom MD simulations, during which some anions dissociated from the initial binding site S1 into site S2 of the large permeation cavity of the protein (Fig. S2) or into the surrounding solution because of the OF nonoccluded conformation of hAE1 and hNBCe1 used in the simulations. The Na^+^ and substrate residence times in the permeation cavity of hAE1 and hNBCe1 are listed in Table 1. In the cases of Table 1 where the initially bound Cl^−^ was substituted with a Cl^−^ from the solution, the Cl^−^ residence times and the frequency of Cl^−^ entry are presented in Figure S4. The final anion and Na^+^ binding sites were established from ion density maps (Fig. S6) and ion–protein contact frequency analysis (Fig. S7). Figure S6 displays the position of the anion and Na^+^ densities (in cyan and yellow, respectively) in the three SLC4 systems with binding stoichiometry corresponding to the previously published functional measurements (a single Cl^−^ or HCO_3_^−^ for hAE1 (7) and Na^+^ and CO_3_^2−^ for hNBCe1 (26)). The ion–protein contact frequencies for these three systems are presented in Figure S7 with a 5 Å cutoff for the ion–protein interactions. Two independent 250 ns MD trials were performed for hAE1 loaded with a single Cl^−^ or HCO_3_^−^ (Table 1), and the anions remained in site S1 in one of the trials and migrated to site S2 in the other trial. Thus, the contact frequencies for hAE1 + Cl^−^ and hAE1 + HCO_3_^−^ in Figure S7 display cumulative results from both independent trials and reflect anion contact with the residues of both sites S1 and S2. The refined binding sites of the substrates in hAE1 and hNBCe1 based on the calculated ion density and contact frequency maps are shown in Figure 3.

### Functional mutagenesis data

**Figure 4:**
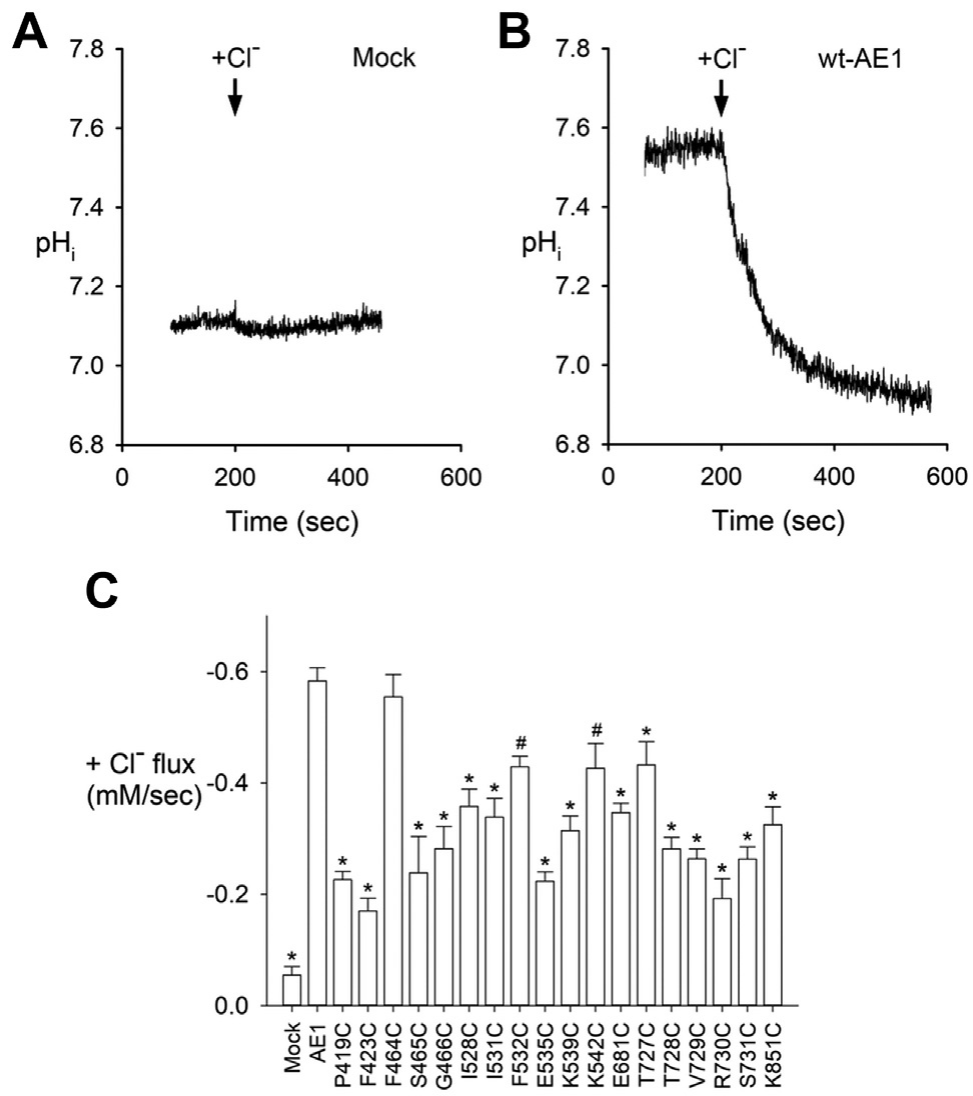
**Cl^−^-driven base transport transients**. *A*, in mock transfected cells. *B*, in cells transfected with WT hAE1. *C*, a summary of the Cl^−^-driven base flux results in the various constructs. One-way ANOVA and Dunnett’s test were used to compare multiple study group means with WT hAE1. Statistically significant results differing from WT hAE1 are depicted with ∗*p* < 0.001 and #*p* < 0.005. Results are depicted as mean ± SEM. See Table S1 for values of n. hAE1, human anion exchanger 1; hNBCe1, human electrogenic sodium bicarbonate cotransporter 1.

**Figure 5:**
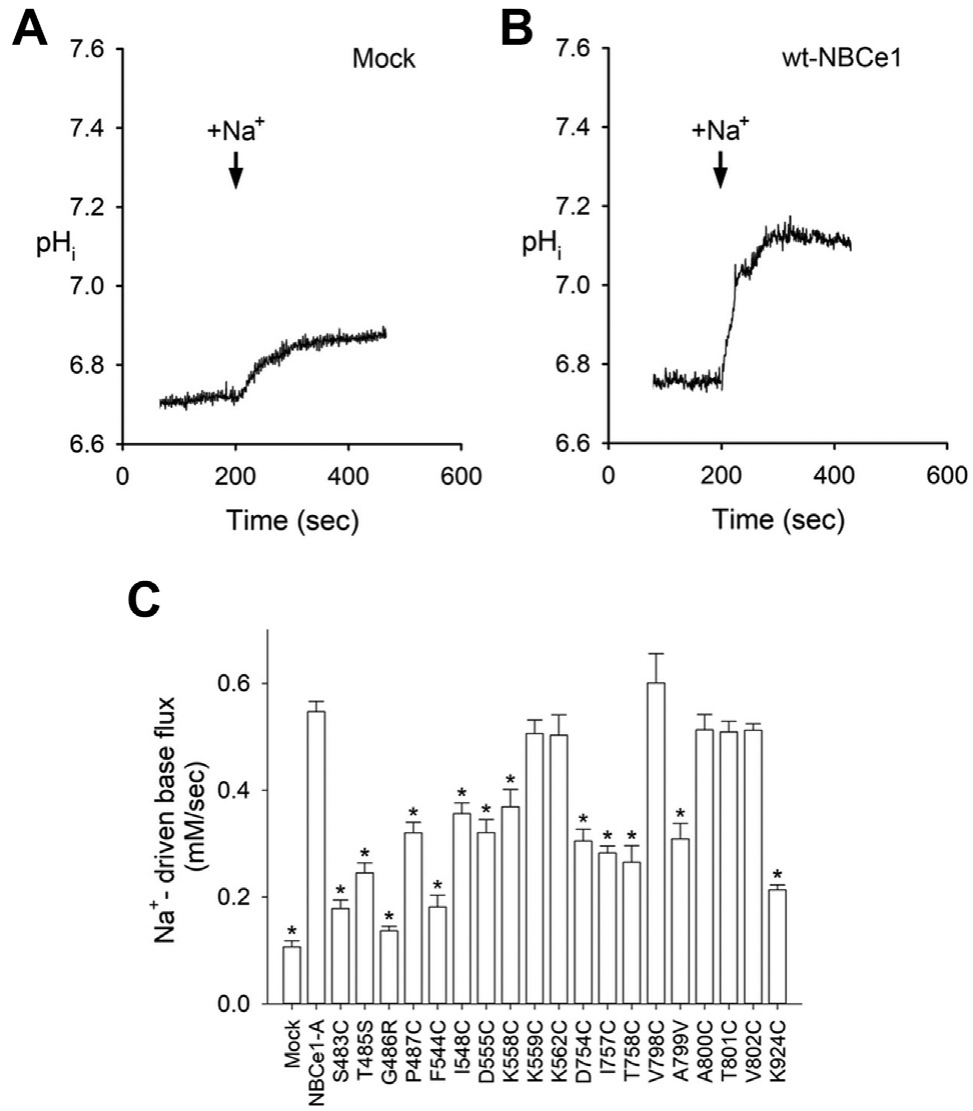
**Na**^**+**^**-driven base transport transients.***A*, in mock transfected cells. *B*, in cells transfected with WT hNBCe1-A. *C*, a summary of the Na^+^-driven base flux results in the various constructs. One-way ANOVA and Dunnett’s test were used to compare multiple study group means with WT hNBCe1-A control group. Statistically significant results differing from WT hNBCe1-A are depicted with ∗*p* < 0.001. Results are depicted as mean ± SEM. See Table S2 for values of n. hAE1, human anion exchanger 1; hNBCe1, human electrogenic sodium bicarbonate cotransporter 1.

Experimental confirmation of the functional significance of the residues outlined in Figure 3 was done with functional mutagenesis and Cl^−^ and Na^+^ driven flux measurements in a number of hAE1 and hNBCe1 single point mutants expressed in Human Embryonic Kidney cell line 293 (HEK293) cells (Figs. 4 and 5, respectively). Figure 4, *A* and *B* shows the Cl^−^-driven base transport transients in mock transfected cells, and cells transfected with WT hAE1. Figure 5, *A* and *B* shows the Na^+^-driven base transport transients in mock transfected cells and cells transfected with WT hNBCe1. A summary of the Cl^−^-driven base flux results and the Na^+^-driven base flux results in the various constructs are shown in Figures 4*C* and 5*C*, and Tables S1 and S2, respectively. Sulfo-NHS-SS-biotin membrane labeling and immunoblotting show comparable levels of membrane expression for all studied mutants to the respective WT hAE1 or hNBCe1 species (Figs. S8 and S9). Mutation to cysteine of most studied residues in hAE1 and hNBCe1 decrease the anion uptake to ~25 to 65% of the WT uptake values (Figs. 4*C* and 5*C*), confirming the functional significance of these protein regions.

## Discussion

### Modeling approach to the binding site identification in the SLC4 family

Computational modeling provides indispensable complementary information to many experimental structural biology and bioinformatics techniques (28, 29, 30, 31, 32). In many cases, the resolution of the X-ray or cryo-EM structures is not sufficiently high to reveal molecular details of the ion/substrate binding sites. Furthermore, the state-specific structures of membrane proteins often afford limited information about the potential access pathways for ions and substrates. A natural solution to this challenge is to combine transport measurements, protein sequence comparisons, and/or structural and molecular modeling (33, 34) using other protein families or within the SLC4 family. This comprehensive experiment-modeling approach aids in the targeted validation of the putative binding pockets and establishes protein–ion/substrate interactions of specific functionally critical amino acid residues (35, 36).

As a first step toward the identification of putative binding sites in hAE1 and hNBCe1, we applied the recently developed SILCS protocol for identification of accessible protein regions capable of binding of anionic and cationic molecular fragments (19). The SILCS approach makes use of a set of predeveloped molecular fragments (probes) to couple sampling of receptor flexibility with Monte-Carlo mapping of the putative binding pockets. To that end, GCMC simulations with oscillating chemical potentials are used for insertion, rotation, translation, and deletion of molecular fragments in the 3D receptor map generated from the all-atom MD simulations of the solvated protein-membrane system in the presence of high fragment concentrations (250 mM per fragment in a system with nine fragments). The excess chemical potentials for the existing library of molecular fragments were developed to accurately reproduce the desolvation penalty for each fragment (19). The SILCS protocol allows rapid “flooding” of the protein-membrane system with fragments (with explicit inclusion of receptor flexibility) and identification of hidden protein binding sites and permeation pathways as high fragment density regions (24, 37).

To expand further our understanding of the protein–ion interactions in the SLC4 family, we performed 1.2 μs all-atom MD simulations to assess the behavior of the relevant physiological ions (Na^+^, Cl^−^, and HCO_3_^−^) in the permeation cavity of apo-hAE1 at near-physiological concentrations. These results were complemented by a set of 250 ns MD simulations with different ion loads in the central site S1 in hAE1 and hNBCe1 (identified from electrostatic mapping of the protein center, SILCS simulations in both proteins, and the 1.2 μs MD simulations in apo-hAE1) which provided additional refinement of the ion binding sites in the permeation cavity and at the center of the protein where the ions have to bind before translocation. Multiple binding events were observed during the MD simulations in line with previously outlined solute behavior in open and nonoccluded structures of ion channels and secondary transporters (38, 39). It is important to stress, however, that the scope of this modeling work is on the ion binding preferences of the OF state in the SLC4 family. The formation of the fully loaded occluded state and the eventual substrate translocation may require significant conformational changes and may involve a complex sequence of events including ion binding to allosteric sites, ion-induced changes in protonation, or formation of additional hydration patterns, destabilizing the OF state of the transporter (40, 41). The complexity of the full SLC4 transport cycle cannot be captured in all-atom MD runs of the lengths reported here and will remain a next frontier for the field. Thus, the main goal of the modeling studies presented here is to identify a set of critical residues involved in ion access and binding to the binding pockets mapped from SILCS simulations and then test these residues experimentally. Accordingly, the residues comprising the proposed binding sites were investigated and confirmed to be functionally relevant by electrophysiological measurements on cysteine mutants of the amino acids implicated in ion–protein interactions from our calculations. Such combinations of computational modeling techniques which bridge and complement various experimental structural biology methods (*e.g.,* X-ray and cryo-EM techniques, functional mutagenesis, evolutionary amino-acid sequence considerations) are routinely applied nowadays in the field of protein-aided membrane transport (28, 30).

### Computations suggest presence of two high affinity substrate binding sites with potentially different functional roles in OF hAE1 and hNBCe1

Both hAE1 and hNBCe1 feature a large well-hydrated OF permeation cavity framed by a number of charged residues, with the positively charged residues outnumbering the negatively charged ones (Figs. 1 and S2). The cavity is lined with basic residues that are attractive to anions. Accordingly, our simulations consistently show areas of high anion density in the permeation cavities of hAE1 and hNBCe1 (Figs. 1, 2, and S6) and frequent permeation of anionic fragments and physiological anions toward the center of the protein (Figs. 1, 2, S1, S3, and S4). In particular, two well-defined areas of high anion density can be discerned in the permeation cavity of hAE1 (Figs. 2 and S6), from our 1.2 μs and 250 ns MD simulations, which correspond to two putative substrate binding sites, labeled here as S1 (central binding site) and S2 (entry binding site). Similarly, the 250 ns MD simulations of hNBCe1 reveal the existence of two anion binding sites, analogous to sites S1 and S2 in hAE1 (Fig. S6). One or both of the sites in hAE1 and hNBCe1 are consistently occupied by anions in most of our MD trajectories suggesting the existence of two high-affinity/high-accessibility substrate binding sites during the substrate translocation dynamics in the OF state of the SLC4 transporters. In the cases where one of the original anions (Table 1) exits the binding cavity during the MD simulation, a Cl^−^ ion from the surrounding solution often takes its place in the cavity (location of site S2 in hNBCe1) or the protein center (location of sites S1 and S2 in hAE1), Figures S1 and S4. The anion access to the positively charged residues in site S2 is not hindered by screening hydrophobic amino acids in the wide, well-hydrated permeation OF cavity of hAE1 and hNBCe1, and considering the frequency of anion entry in this site, it likely serves as a transient binding site whose function is to attract anions from the extracellular solution to the cavity before their movement to the central site S1, where anion binding triggers the protein reorganization required for a transport event. The two binding sites with the relevant substrates are presented in Figure 3 for hAE1 loaded with one HCO_3_^−^ or one Cl^−^ (from two independent 250 ns MD trials of hAE1+HCO_3_^−^ and hAE1+ Cl^−^ where binding at both sites S1 and S2 occured), hNBCe1 loaded with one Na^+^ and one CO_3_^2−^ (for illustration of stable ion pair binding at site S1) and hNBCe1 loaded with one CO_3_^2−^ (for illustration of binding at site S2 after the CO_3_^2−^ ion exited from site S1).

Previous electrophysiology studies provided clues for the existence of more than one substrate binding sites (*i.e.,* transfer and modifier sites) at the intracellular and extracellular side of hAE1 (10). Some of these sites (approach sites) have been implicated in redirection of the approaching substrates toward or away from the transport-triggering substrate binding site (alternating site) and could be involved in the experimentally observed self-inhibition of hAE1 at high substrate concentrations (10, 42). The two sites identified here are compatible with the suggested model of an alternating (site S1) and an approach site (site S2). Future studies will ascertain the specific mechanistic roles of the newly identified sites S1 and S2 and their involvement in various competitive inhibition and self-inhibition scenarios.

### Anion binding sites in hAE1: Site S2

The entry binding site S2 of hAE1 is occupied by an anion in most of our MD simulations. Site S2 is comprised of residues P419, F423, F464-G466, I528, V531, F532, E535, K539, T727, V729, R730, and K851 (Fig. 3), which are found with highest frequencies within 5 Å of the Cl^−^ or HCO_3_^−^ ion in this site (Fig. S7). These residues are highly evolutionary conserved (ConSurf-DB (43) scores 8–9, Fig. S10). In accordance with the ConSurf-DB scores, mutations to cysteine of all S2 residues except F464 have significant detrimental impact on hAE1 transport properties (Fig. 4*C*) without impacting protein expression levels in the membrane (Fig. S8) confirming the ion transport relevance of this protein area. As evident from Figure 3, the side chains of all S2 residues except F464 are oriented toward the HCO_3_^−^ or Cl^−^ anion and participate in its coordination. Mutation of these side chains to cysteine is therefore expected to alter significantly the protein function, consistent with our experimental observations of 35 to 70% flux decrease (Fig. 4*C*). Anion coordination by residue F464 is achieved *via* its protein backbone atoms. Single point substitution at position F464 with smaller side chains, such as cysteine therefore would be expected to have a less profound effect on hAE1 transport (as observed in Fig. 4*C*, where the F464C mutant exhibits almost 100% of the WT activity), as long as helical packing and electrostatics in the region are not heavily affected.

Site S2 shows direct involvement in bicarbonate coordination of residue E535 (*via* the proton of HCO_3_^−^), which is one of the acidic residues in the binding pocket of hAE1 (Fig. 3). Accordingly, E535C mutation leads to drastic decrease of hAE1 transport (Fig. 4*C* and Ref. (13)). Our 1.2 μs MD simulations of hAE1 in equimolar solution of HCO_3_^−^ and Cl^−^ ions demonstrate that the two anions compete for site S2 with HCO_3_^−^ outcompeting Cl^−^ and binding more often (higher number N of unique entry events, Fig. S1*A*) and for longer periods of time (average residence times of 151.17 ns *versus* 3.96 ns, respectively, Fig. S1*A*) to site S2 in the protein center. The trigonal planar geometry of HCO_3_^−^ allows for a good overlap of the carbonate group with the guanidinium group of R730 and additional coordination with K539 with a formation of a H-bond between HCO_3_^−^ and E535 (Fig. 3) in site S2. Thus, in the presence of HCO_3_^−^, the residence time for Cl^−^ decreases considerably (average residence times in the protein center of 24.44 ns *versus* 3.96 ns, for hAE1 in pure NaCl and hAE1 in equimolar mixture of NaCl and NaHCO_3_, respectively) because of the competition between HCO_3_^−^ and Cl^−^ for site S2. From a functional point of view, this enhanced binding pattern between glutamate and HCO_3_^−^ (Fig. 3) may manifest as a selective preference of OF hAE1 for HCO_3_^−^ (glutamate at this position is featured in SLC4A7, 8, 10, where the transported substrate is likely a HCO_3_^−^ ion) which is found in ~4-fold lower concentration in the extracellular fluid than Cl^−^ (25).

Although the presence of a negatively charged residue in an anion binding site seems counterintuitive, it should be noted that the permeation cavity of hAE1, where site S2 is situated, is lined also with four positively charged residues (K539, K542, R730, and K851, Figs. S2 and 3) which overcome the repulsion effect of E535 and facilitate attraction of the anion substrates from the surrounding solution to site S2 (Figs. 1, 2, S1, and S3), implying that the role of this site is to draw substrate anions from the extracellular space toward the protein center in preparation to binding to the central site S1. With the exception of K542 (ConSurf-DB score 2), all these positively charged residues are highly conserved (Fig. S10). The importance of the lysine residues in site S2 has been assessed in previous works with contradicting results (44, 45, 46, 47). Our functional mutagenesis data show that neutralization by cysteine substitution of each lysine residue of site S2 leads to 30 to 50% decrease in Cl^−^ driven base flux compared with WT (Fig. 4*C*), reinforcing the notion that these residues are important participants in the hAE1 transport function. In addition, K539 and K851 are the anchor points for the covalently bound 4, 4′-diisothiocyanatostilbene-2, 2′-disulfonic acid (DIDS) molecule, which blocks hAE1 transport and locks the transporter in the OF state (48, 49, 50, 51, 52).

### Anion binding sites in hAE1: Site S1

The central binding site S1 of hAE1 is formed by residues I528, F532, and the T727-S731 stretch, all of which are key for hAE1 transport, according to our functional mutagenesis results (Fig. 4*C*), previous studies (13), and evolutionary conservation deliberations (ConSurf-DB scores 8–9, Fig. S10). Considering the proximity of site S1 to the protein center, at the junction point of the catalytically relevant portions of TM3 and TM10 (6, 11, 12, 16, 17, 18, 53), it is likely that the occupation of this site is necessary for initiation of the protein conformational changes, which lead to anion translocation.

Our MD simulations in hAE1 emphasize the importance of R730 in coordination of HCO_3_^−^ and Cl^−^ (Fig. 3) supported by the functional mutagenesis and uptake measurements (Fig. 4*C* and Ref. (54)). Cysteine substitution of R730 decreases the observed Cl^−^ flux to ~30% of the WT values (Fig. 4*C*). The R730C mutation causes hereditary stomatocytosis and has a well-established association with impaired anion transport and increased cation leak in hAE1 (54). In our simulations, R730 is responsible for stable binding of all tested anions in hAE1 including CO_3_^2−^, whereas its presence leads to rapid dissociation of Na^+^ from the binding site of hAE1 if CO_3_^2−^ with its stabilizing -2 charge is not present (Table 1), an observation supported also by our SILCS maps in Figure 1 and Na^+^ entry statistics from 1.2 μs MD simulations of apo-hAE1 in Figure S3. The side chain of R730 demonstrates flexibility within the well-hydrated permeation cavity of hAE1 and the rotation of its bulky guanidinium group propels the anionic substrates of hAE1 to move between site S1 and S2 once the substrate anions reach the central part of the protein. When an anion is bound to site S2, the R730 side chain extends upward where it can participate in the S2 binding motif, specifically the charged residues E535, K539, and K851 (Fig. 3). In site S1, R730 “folds” around the anion, and the anion is stabilized by interactions with several backbone NH groups (residues T728 and V729) in addition to the electrostatic attraction to the guanidinium group and the remaining side chains in the vicinity (Fig. 3). The side chain conformation of R730 may therefore have important mechanistic implications for strong binding of the anion before translocation. Its position in the vicinity of the hydrophobic residues of site S1, V729, F532, and I528, whose side chains are oriented toward one another forming a hydrophobic plug at the extracellular side of hAE1 (Fig. 3) preventing water and ion access to the intracellular space, suggests that R730 may be involved also in gating events because of structural reorganizations in this area.

### Protonation of residue E681 in site S1 of hAE1 leads to altered ion dynamics in the protein cavity and center

hAE1 and hNBCe1 are involved in pH regulation and are sensitive to pH changes (1, 8, 46, 55). Protonation/deprotonation of the highly conserved (Figs. S10 and S11) acidic residues in the vicinity of sites S1 and S2 in hAE1 and hNBCe1 could therefore be a part of the pH-dependent transport response of the SLC4 family. In analogy to other secondary transporters (56, 57, 58), there is a number of titratable residues around the ion binding pockets, which may potentially change their protonation states in an ion-dependent manner. PROPKA (59, 60) pKa calculations (at the original protein geometry deposited in the PDB (11, 12)) show that the pKa of the carboxylate group of E681 is elevated significantly compared with the reference value in the aqueous phase (pKa 6.8 for the protein *versus* model pKa of 4.5). To probe whether protonation in the identified site S1 has an impact on ion retention in the binding pocket, we performed a set of 250 ns MD simulations of hAE1 with protonated E681, loaded with either Cl^−^ or HCO_3_^−^ in site S1 (Table 1). Additional 1.2 μs MD simulations were also performed on apo-hAE1 with protonated E681 in a 150 mM NaCl solution and in a 75 mM NaCl +75 mM NaHCO_3_ mixture (Figs. S12–S14). The detailed description of pH dependence of the entire transport cycle in hAE1 and hNBCe1 and how it relates to the newly identified binding sites S1 and S2 would require additional computational and experimental efforts and is outside of the scope of the current work. However some interesting tendencies in ion permeation which hint at pH-dependent transport can be observed from our MD simulations of hAE1 with E681 in protonated and deprotonated forms, respectively.

The residence time of the ions/substrates in these simulations may provide a surrogate metric of binding site accessibility and ion retention as a function of E681 protonation. Protonation in the hAE1 systems leads to frequent entry of more than one anion in the protein cavity and increased incidence of more than one anion in the putative binding sites at the protein center, in comparison to the non-protonated hAE1 systems (Figs. S1, S3, S4, S12, and S13). Upon protonation of E681, the protein center remains consistently occupied by at least one anion for the duration (250 ns and 1.2 μs) of our MD simulations, with Cl^−^ and HCO_3_^−^ ions from the extracellular solution permeating to the protein center and exchanging with the anions bound there (Figs. S1, S4, and S12). Moreover, a sizable anion density produced from the 1.2 μs MD trajectories in the apo-hAE1 protonated at E681 is observed in both sites S1 and S2 (Fig. S14), implying that protonation of this residue might be required for the stable anion binding to site S1 preceding the anion translocation event.

E681 located at the protein center of hAE1 was identified as a plausible proton-binding site during H^+^-sulfate cotransport in hAE1 (61). It was shown that E681 is a crucial residue in hAE1 for Cl^−^-mediated anion exchange (Cl^−^ flux decreases with ~40% in E681C mutants, compared with WT hAE1, Fig. 4*C*) although it falls outside of the 5 Å cut-off for protein residues, presented in Figure S7. Previous studies suggest that E681 plays a role both in substrate selectivity and pH sensitivity of AE1 (62). The chemical modification of the glutamate carboxyl group to alcohol with the Woodward’s reagent K inhibits the anion exchange (63). Mouse E699Q mutation (E699 is homologous to human AE1 E681) has a strong impact on Cl^−^/HCO_3_^−^ exchange (64) similar to the hAE1 E681C mutation (65) (Fig. 4*C*). This implies that successful function of hAE1 requires a glutamate residue at position 681, because even the conservative E681D mutation leads to significantly lowered AE1 exchange activity (62). Thereforе, the combination of previous experimental studies and the modeling studies presented in our work endorse а transport mechanism in which protonation at the position E681 might be important for effective anion transport in hAE1.

### Anion binding sites in hNBCe1: Site S2

The anion maps in the 250 ns MD simulations of hNBCe1 also indicate two putative binding sites (central site S1 and entry site S2) in the areas of high anion excess density from the hNBCe1 SILCS maps (Fig. 1), in analogy to the ones observed in hAE1. Site S2 in hNBCe1 is positioned within the cavity region defined in Figure S2, further away from the protein center than site S2 in hAE1 because of the absence of a positively charged residue analogous to R730 in the protein center of hNBCe1, which would draw the anions deeper in the cavity. Site S2 consists of a cluster of lysine residues (K558, K559, K562) and an aspartate residue (D555) which is the hNBCe1 analog of E535 in hAE1 (Fig. 3). Residues K558 (ConSurf-DB score 6) and K562 (ConSurf-DB score 2) are less evolutionary conserved than residues D555 and K559 (ConSurf-DB scores 9), Figure S11. The function of site S2 in hNBCe1 is similar to the one observed in hAE1—a transient binding site, which serves as an attractor of anions from the surrounding solution. The anion and cation dynamics in the cavity region of hNBCe1 from a 250 ns MD simulation of apo-hNBCe1 in 150 mM Na_2_CO_3_ (Fig. S15) and hNBCe1 in 150 mM NaCl (Figs. S4 and S15) bears some resemblance to the one observed in hAE1 (Figs. S1 and S3) with anions permeating more often in the cavity than the Na^+^ ions and residing in the area of the entry site S2. The divalent CO_3_^2−^ has longer residence times at the cluster of lysine residues (K558, K559, K562) of site S2 than Cl^−^, and the majority of MD steps feature a CO_3_^2−^ bound in this area (78.07% of MD steps for CO_3_^2−^ *versus* 17.23% for Cl^−^, Fig. S15). The anions did not reach the center region of hNBCe1 where site S1 is located during the 250 ns MD simulations because of the absence of a positively charged residue analogous to R730 in hAE1 in this area that would attract them deeper in the cavity. Na^+^ entry in the cavity region of hNBCe1 is hindered by the screening effect of the lysine residues in site S2, and no Na^+^ ions were found in the protein center.

Na^+^-driven base flux comparable to WT hNBCe1 is recorded for the cysteine substitutions of K559 and K562 (Fig. 5*C*) from site S2, which are positioned further away from the protein center than K558 and D555, and their long flexible side chains move freely and often stray away from site S2 (Fig. 3). Substitution to cysteine of K558 and D555, which are closer to the protein center decreases the Na^+^-driven base flux in hNBCe1 to less than 60% of the WT values. The functional mutagenesis results for site S2 in hNBCe1 do not feature the clear correlation between the ConSurf-DB scores and transport impairment which was evident in hAE1 (see above). Previous studies on the lysine residues of site S2 in hNBCe1 are also inconclusive about the mechanistic significance of these residues: K559 has been identified as important for reversible DIDS blockade, whereas K558, K559, and K562 are not essential for irreversible DIDS blockade (66). However, cysteine substitution of the highly conserved D555 of site S2 has a profound negative effect (more than 40% decrease with respect to WT) on Na^+^-induced base flux (Fig. 5*C*). A D555E substitution in hNBCe1 induces a Cl^−^ flux (67), which implies potential role in substrate selectivity of residue D555 in hNBCe1 and more generally (considering our observations for preferred HCO_3_^−^ binding to residue E535 in hAE1), the acidic residues at this position in the SLC4 family. The sodium-dependent chloride bicarbonate exchanger (SLC4A8) also features a glutamate residue in the area of site S2 (8). Thus, the acidic residue in site S2 potentially provides structural basis for discrimination not only between the substrates/co-permeant ions of hNBCe1 and hAE1 (Na^+^ + CO_3_^2−^ *versus* Cl^−^) but also between the two substrates of hAE1 (Cl^−^ *versus* HCO_3_^−^).

### Anion binding sites in hNBCe1: Site S1

The central site S1 of hNBCe1 is comprised of S483, T485-P487, F544, I548, D754, I757, T758, V798-V802, and K924 (Fig. 3), all of which are highly evolutionary conserved (ConSurf-DB scores 9, Fig. S11). Our functional mutagenesis data demonstrate that single point mutations to cysteine or to residues implicated in proximal renal tubular acidosis (T485S, G486R, and A799V (68, 69)) of the amino acids of site S1 decrease hNBCe1 transport to 35 to 60% of the measured WT values in the majority of the studied mutants (Fig. 5*C*), confirming the transport relevance of these residues. Site S1 residues that have seemingly little impact on transport are the residues from the V798-V802 stretch which participate in ion coordination *via* their backbone NH (CO_3_^2−^ coordination) or CO groups (Na^+^ coordination) (Fig. 3). Substitution with a smaller slightly polar residue such as cysteine at these positions has a negligible effect on the ion/substrate coordination (similarly to the F464C mutation in hAE1). The introduction of a bulkier side chain in the pRTA mutation A799V (68) however may disturb the geometry of site S1 enough to warrant the observed more significant decrease in transport (Fig. 5*C*). The polar residue T801 is involved in CO_3_^2−^ coordination also through its hydroxyl group. This coordination may be recaptured by the thiol group of cysteine in the T801C single point mutant, leading to the observed negligible deviation from the Na^+^-driven base flux values of WT hNBCe1 (Fig. 5*C*). From the residues whose mutation has more considerable effect (flux decrease with 40% and more compared to WT values) on hNBCe1 transport, residue T485 has shown clear functional significance, because the conservative serine substitution (which is also a pRTA mutation (68)) at this residue leads to decrease in Na^+^-driven base flux and nonelectrogenic carbonate transport (26). Another pRTA mutation, G486R (69), shows highly impaired hNBCe1 transport (less than 20% of WT function, Fig. 5*C*). Considering the immediate exit of Na^+^ placed without the stabilizing CO_3_^2−^ ion in the binding site of hAE1 (Table 1, hAE1+Na^+^), the presence of an arginine residue in the binding site of hNBCe1 most likely would impede Na^+^ binding leading to the observed transport deficiency.

### Sodium binding in hNBCe1

The Na^+^ ion in site S1 of hNBCe1 is coordinated by the carboxyl group of D754, the hydroxyl group of T758, and the backbone carbonyl oxygen of A799 in addition to the CO_3_^2−^ ion (Fig. 3). Residue D754 which we identify as the main Na^+^ binding residue in site S1 of hNBCe1 both from SILCS mapping (Fig. 1, right) and MD simulations (Fig. 3), is analogous to residue E681 in hAE1. Our functional mutagenesis data and previous studies (65) show that hNBCe1 transport decreases significantly (with ~50%) upon D754C substitution (Fig. 5*C*). Site S1 of the non-protonated WT hNBCe1 systems exhibits stable substrate coordination, which persists for more than 250 ns only when Na^+^ and CO_3_^2−^ are present together (Table 1). All other studied binding stoichiometries are not stable leading to rapid substrate dissociation from site S1 into site S2 or the surrounding solution. This implies that in the absence of a positively charged residue in a position similar to R730 in hAE1, a Na^+^ ion may assume the role of a positively charged residue in the central binding site of hNBCe1.

All known Na^+^-dependent SLC4 transporters (SLC4A4, 5, 7–10) feature an aspartate residue at this position, whereas the Na^+^-independent anion exchangers (SLC4A1-3) and the H^+^-NH_3_ transporter SLC4A11 have a conserved glutamate residue. Na^+^ binding is very sensitive to protonation at residue D754 (evaluated PROPKA pKa values 5.23, with reference pKa value for aspartate in water 3.8 (60)). The protonation of D754 leads to swift dissociation of Na^+^ ions followed by immediate displacement of the CO_3_^2−^ ion toward the exit of the OF permeation cavity and site S2 (Table 1). Based on their pKa values, at the physiological pH~7, E681 of hAE1 has a higher likelihood to be protonated than D754 of hNBCe1. The presence of an aspartate residue at this position in the Na^+^-dependent SLC4 transporters may therefore be an evolutionary necessity for maintenance of Na^+^ binding at physiological pH values, because Na^+^ appears to be critical for stable coordination of the anion substrate in the central site S1 in the absence of a positively charged residue like R730 (Table 1).

SILCS methylammonium maps of the permeation cavity of hNBCe1 indicate that Na^+^ may be able to traverse the cavity and bind at residue D754 (Fig. 1). However, in most of our 250 ns MD simulations of hNBCe1, in the absence of CO_3_^2−^, the Na^+^ ions do not reenter the cavity after dissociation from their initial position in site S1 (Table 1). To assess the frequency of Na^+^ entry in the hNBCe1 cavity we ran a 250 ns MD simulation of apo-hNBCe1 with 150 mM Na_2_CO_3_ and compared these results to hNBCe1 in 150 mM NaCl, where no CO_3_^2−^ ions are present in the solution (Fig. S15). The Na^+^ entry in the lysine rich cavity of hNBCe1 occurs in less than 10% of the MD steps; however, there is a small 5% increase of the instances in which 1 Na^+^ can be found in the cavity when CO_3_^2−^ ions are present in the area of site S2 (in the hNBCe1 in Na_2_CO_3_ solution, where the majority of the MD steps feature a CO_3_^2−^ bound to site S2). The presence of CO_3_^2−^ in site S2 draws Na^+^ ions from the extracellular solution, which form short lived ion pairs with the bound CO_3_^2−^ (Fig. S15). Thus, CO_3_^2−^ binding at site S2 may be a necessary first step for Na^+^ to overcome the electrostatic repulsion from the positively charged lysine residues in the vicinity of site S2 and for the successive translocation of both Na^+^ and CO_3_^2−^ toward the protein center and its binding site S1 (Fig. 3). Considering the lack of entry of anions to the protein center in hNBCe1 (Fig. S15) and the stable coordination of a Na^+^-CO_3_^2−^ ion pair in site S1 of hNBCe1 (Table 1), binding of a Na^+^ ion to site S1 might be a necessary precursor for CO_3_^2−^ migration to site S1. Alternatively, both ions could migrate from site S2 to site S1 together as an ion pair. We did not observe such migration events in the 250 ns duration of our hNBCe1 MD simulations. Interestingly, in the Na^+^-dependent nonelectrogenic symporters of the SLC4 family (SLC4A7, 10), the analogous residues to K558 and K562 are substituted with acidic residues leading to a cluster of three negatively charged amino acids in the area of site S2 (8). This is expected to facilitate permeation of Na^+^ in the cavity to a larger extent than the observed in our MD simulations of hAE1 and hNBCe1, even in the absence of an anion at this position.

In conclusion, we combine several complementary computational techniques with functional mutagenesis measurements to probe the putative binding pockets in hAE1 and hNBCe1 and to identify substrate binding sites and specific functionally relevant ion–protein binding motifs. Our MD simulations are in good agreement with our functional mutagenesis data and previous functional studies in the SLC4 family and provide novel insights into the transport mode determinants (Na^+^-dependent *versus* Na^+^-independent transport) in the SLC4 family. We also highlight potential new avenues for future investigations, including specific mechanistic roles of the newly identified sites S1 and S2, role of individual residues in sites S1 and S2 in substrate selectivity and ion binding, role of protonation of the acidic residues at the protein center in substrate selectivity, binding and kinetics of transport, and role of the side-chain of residue R730 and adjacent nonpolar residues at the protein center in ion binding and gating. Better understanding of the substrate binding sites in SLC4 and their involvement in the overall transport process opens the door to design of specific and non-specific activators and inhibitors of SLC4 transport, which can be potentially applied in the treatment of SLC4-related diseases.

## Experimental procedures

### hAE1 and hNBCe1 model preparation

Unless specified otherwise, the hAE1 and hNBCe1 models used in the computational portion of the manuscript were prepared as follows. The 3D structures of the membrane domains of hAE1 (PDB ID 4yzf) and hNBCe1 (PDB ID 6caa) were taken from Refs. (11, 12), and missing residues were added with Modeller 9.18 (70). The simulation models were built with the Chemistry at Harvard Macromolecular Mechanics (CHARMM)-GUI server (71, 72, 73). Although hAE1 and hNBCe1 are expressed as dimers, their monomers are likely functionally independent, in parallel with other secondary transporters of the same architecture (53). Thus, the simulated systems consisted of a single hAE1 or hNBCe1 monomer, embedded in a 1-Palmitoyl-2-oleoylphosphatidylcholine (POPC) membrane (138/131 POPC molecules in upper/lower leaflet of hAE1 and 156/150 POPC molecules in upper/lower leaflet of hNBCe1, respectively), 20 Å water layers on both sides of the bilayer, and 150 mM NaCl solution. Periodic boundary conditions were implemented with initial periodic box dimensions of 106.5 × 106.5 × 111.5 Å and 112.4 × 112.4 × 138.0 Å for the hAE1 and hNBCe1 models, respectively.

### SILCS mapping of putative cation and anion binding pockets in hAE1 and hNBCe1

SILCS Software (Site Identification by Ligand Saturation, version 2020.1) (37, 74, 75, 76, 77, 78) was used for preliminary identification of putative Cl^−^/HCO_3_^−^ and Na^+^/CO_3_^2−^ ion binding sites in hAE1 and hNBCe1. SILCS is implemented in the Gromacs MD simulation package (79, 80, 81) and makes use of GCMC/MD methodology in oscillating μ_ex_ (excess chemical potential) simulations (76) to yield “FragMaps” for small molecular fragments with different physical and chemical properties that cover the whole protein. The method relies on the fact that the average μ_ex_ of each solute from the simulations approximate the hydration free energy of the solute. Ultimately, extensive solute sampling inside/around the proteins in an aqueous media is achieved which in turn yields information on the mostly likely locations of the probe molecules (solutes) that the target proteins favor. One of the major advantages of the SILCS methodology is that by incorporating MD simulations to GCMC computations it takes into account protein flexibility in identifying the possible ligand-targeted regions which improves conformational sampling of the proteins in addition to the configurational sampling of the probe species. FragMaps are free energy-based 3D-maps (GFE Maps) representing the occupancy possibilities (binding affinity) of small probe molecules/ions that target the specific volumes of the relevant proteins. The SILCS GCMC/MD protocol utilizes benzene, propane, methanole, formamide, imidazole, methylammonium, methanole, acetaldehyde, and acetate species. In the current work, we make use of a Methylammonium N Map which represents the regions that are likely to be targeted by positively charged donor fragments (used as a surrogate for the physiologically relevant Na^+^ ions) and an Acetate O Map, which represents the putative binding sites for negatively charged donor fragments (and by extension, the physiologically relevant HCO_3_^−^, CO_3_^2−^, and Cl^−^ anions). Other maps (benzene and propane as probes for aromatic and aliphatic properties in Generic Apolar Map; formamide and imidazole polar nitrogens as neutral donors in Generic Donor Map; methanole, formamide, and acetaldyhde polar oxygens and imidazole polar nitrogen as neutral acceptors in Generic Acceptor Map; a water map for the identification of favorable water locations and exclusion maps where neither fragments nor waters can be found) were also generated but are not discussed in the current work. The simulation protocol employed here is described below.

#### SILCS system set-ups and equilibrations

Equilibrated frames from 250 ns MD simulations (see below) were used as the starting structures for the SILCS feeds. Gromacs MD (version 2018) (79, 80, 81) was used for the simulations. Bare proteins were embedded in a x:y dimensions of a 120:120 Å POPC (palmitoyloleoylphosphatidylcholine)-cholesterol mixture of 9:1 composition representing the lipid membrane. Afterwards, ten different protein+lipid+water+fragment systems were prepared by solvating the protein–lipid complex with TIP3P waters (82) and the fragments mentioned above. The configurations of waters and fragments were randomly distributed for each of the ten systems after avoiding the overlap of the molecules. Throughout the simulations, the CHARMM 36 (83, 84, 85) all-atom force field for proteins, lipid and water and the CHARMM General Force Field (CgenFF) and CGenFF programme (version 2.3.0) (86, 87, 88) for fragments were used. The molarity of each of the nine fragments and the water phase were set to 0.25 M and 55 M, respectively. The energy of the systems was minimized with 5000 steps steepest descent algorithm. Then six-step equilibration MD simulations were performed on the systems at 300 K. LINCS algorithm (89) was used in the minimization and equilibration steps to constrain the hydrogen bonds. Lennard-Jones interactions were handled with the Verlet cut-off scheme with Force-switch option at 0.5 to 0.8 cut-off range. Columbic interactions were treated with the Particle-Mesh-Ewald technique using a value of 0.8 for the cut-off distance. The first two stages of the equilibrations were realized in a MD ensemble with constant number of particles (N), volume (V), and temperature (T) with Berendsen thermostat coupling (90) for 50,000 steps in each stage. Afterward, in the third, fourth, and fifth stages, MD ensemble with constant number of particles (N), pressure (P), and temperature (T) (NPT) simulations were done at 1 bar maintained by Berendsen thermostat and semiisotropic barostat (90) with 4.5e-5 compressibility factor. In the last step, the thermostat and barostat were switched to Nose-Hoover (91, 92) and Parrinello-Rahman (93, 94), respectively, whereas the other settings remained the same as in the previous isothermal-isobaric simulations. In the first three steps of the equilibration runs, we employed 1 fs timestep for 50,000 steps, whereas for the remaining steps we employed 2 fs timestep for 100,000 steps. Throughout the equilibration steps, harmonic force constants on the protein backbone, protein sidechain, and lipid heavy atoms were gradually decreased. Subsequent to this six-step-restrained dynamics, 5 ns production MD runs with 2 fs timestep were generated by imposing 50.208 kJ mol^−1^ nm^−2^ harmonic force constant on the C_α_ atoms of the proteins. In this step, temperature and pressure were maintained at 300 K and 1 bar by means of Nose-Hoover and semiisotropic Parrinello-Rahman controlling schemes. LINCS algorithm was used to constraint the hydrogen bonds. The approximate number of atoms of each simulated systems was ~90,000.

#### SILCS GCMC and GCMC/MD simulations

Prior to the actual GCMC/MD simulations, 25 sequential steps of GCMC computations were realized in the absence of MD simulations. The last frames of the previous equilibration stages were used as the initial structures for each of the GCMC steps. Each step involved 200,000 MC steps where four scenarios of the water and probe molecules were attempted: insertion, deletion, translation and rotation. These scenarios occur in an active subvolume defined inside the simulation box (74). Metropolis criteria were used to determine whether these attempts would be accepted or not (76). Here, the acceptance probabilities depend on three factors: the target concentration of the solute, its excess chemical potential, and the energy change in the systems between two attempts. In these sequential GCMC simulations, two sets of μ_ex_ values alternate in each three-cycles for a better sampling (76). The μ_ex_ sets take different values in each of the ten discrete simulations, producing an identical average μ_ex_ value, eventually. GCMC simulations sequentially proceed *via* this iterative fashion for 25 steps. Subsequently, GCMC/MD hybrid simulations start with the outputs of the previous 25th step of GCMC simulations. In these hybrid simulations, a MD step is inserted between each of two GCMC steps. Hundred GCMC/MD steps were executed in the current work. Two new sets of μ_ex_ values are alternately used in each three iterative GCMC simulation steps where they are calculated according to a predefined scheme where the magnitude of the deviations of the concentrations of the probes from their target concentrations in a cycle is the factor (76). The used MD protocol consists of a 5000 steps stepest descent minimization, followed by a 300 ps equilibration (with V-Rescale (95) and Berendsen temperature and pressure control, respectively) and a 1 ns production run (500,000 steps of 2 fs each) in a NPT, yielding a total simulation time of 1 μs (10 × 100 ns). In the production stages, C_a_ atoms of the proteins were restrained with 50.208 kJ mol^−1^ nm^−2^ harmonic force constant. Temperature and pressure were maintained at 300 K and 1 bar with Nose-Hoover and semiisotropic Parrinello-Rahman controlling schemes. Hydrogen bonds were constrained with the LINCS algorithm.

#### SILCS FragMaps generation

FragMaps were generated from the 100 steps GCMC/MD simulations where 100 frames were collected from each step (each 10 ps from the production stages). A grid spacing of 1 Å is used in the calculation of FragMaps from the simulation data. First, occupancy maps were generated from each simulation step, and then, selected atom types from the solutes were binned in a 1 Å^3^ voxel volume spanning the whole protein. Then, these occupancy maps were converted to GFE maps (grid free energy map) according to the formula below (75):

$$GFE_{xyz}^{T}=min\left\{-RTlog_{e}\frac{occupancy_{x,y,z}^{F}}{\left\langle bulkoccupancy\right\rangle },3\right\}$$

where GFE of each fragment is calculated in x, y, z coordinates after normalization of its occupancy to its bulk occupancy. GFE maps are capped at 3 kcal/mol. Ultimately, GFE energy values are attained in the voxels that enable the description of the protein binding affinity sites. The convergence of SILCS Fragmaps are judged by overlap coefficient values (75). In the evaluation of overlap coefficient values, the ten discrete simulations are divided into two groups and FragMaps of these subgroups are recalculated to further measure their similarity according to a scheme described in (75). In the current study, all overlap coefficients belonging to the used fragments are above 0.70 which indicates a reasonable convergence of the simulations for both hNBCe1 and hAE1 systems.

### MD simulations of hAE1 and hNBCe1 with different ion loads in site S1

#### Protein:ion stoichiometry considerations

Tentative binding sites and stoichiometry of transport in hAE1 and hNBCe1 were previously established from comparison to other transporters of the same architecture and from functional mutagenesis and electrophysiology studies (11, 12, 15, 16, 17, 18). AE1 features electroneutral transport, which implies that it exchanges equal amounts of HCO_3_^−^ and Cl^−^ (*e.g.,* 1:1, 2:2, etc) (7). The transport by NBCe1 is electrogenic and requires two negative charges co-transported with one positive charge (26, 96). The necessary charge of -2 in this case could be achieved either by a single CO_3_^2−^ ion or by two HCO_3_^−^, whereas the +1 charge is provided by a single co-transported Na^+^ ion. Previous studies have indicated that CO_3_^2−^ is the transported species (26, 27). The size and the amino acid composition of the putative binding sites suggest that accommodating more than one CO_3_^2−^ or HCO_3_^−^ ions, at the same time, close to the protein center (location of site S1 in both proteins), where the ions have to bind to translocate, is unlikely. Thus, the hAE1 and hNBCe1 transport most probably involves a single CO_3_^2−^, HCO_3_^−^, or Cl^−^ ion (7, 26).

#### Starting position of the ions at site S1

To probe the ion–protein interactions at the central site S1, we constructed and subjected to 250 ns MD simulations a number of hAE1 and hNBCe1 models (Table 1) with different ion binding stoichiometries and different protonation states of the central acidic residues (E681 in hAE1 and D754 in hNBCe1) in the vicinity of the putative binding site implicated in determination of SLC4 transport modes (exchange *versus* symport) (12). One or more ions (Table 1) were placed in the binding pocket, in the area of site S1 identified from SILCS mapping and 1.2 μs MD simulations with the apo-hAE1 structures. The position and orientation of the ions were further guided by Poisson-Boltzmann electrostatic maps generated with the Poisson-Boltzmann Equation Module solver of CHARMM-GUI (72, 97) (Fig. S5). Both hAE1 and hNBCe1 feature a large positively charged permeation cavity in the area of site S1, lined with lysine residues, which leads to the center of the protein where the ion binding is expected to take place before translocation is initiated (Fig. S2). A negatively charged area in the protein center resulting from the presence of an acidic residue (E681 in hAE1 and D754 in hNBCe1) may provide sufficient stabilization for positively charged species such as Na^+^ in hNBCe1 (as suggested by SILCS maps of the hNBCe1 OF cavity, Fig. 1) or the proton of HCO_3_^−^ in hAE1 (Fig. S5). The basic R730 residue in the center of hAE1 is an obvious coordinating ligand for negatively charged species like Cl^−^, HCO_3_^−^, or CO_3_^2−^. The hNBCe1 protein lacks a positively charged residue analogous to R730 in hAE1. However, the remaining residues in its center create a protein surface with a weak positive charge that could potentially attract an anion (Fig. S5) as evident from SILCS mapping of the OF hAE1 and hNBCe1 (Fig. 1, right) and ion densities generated from 1.2 μs MD simulations of apo-hAE1 in solutions containing Cl^−^ and HCO_3_^−^ ions (Fig. 2). Following the electrostatic maps, a single anion was added in site S1 of the empty protein (according to the ion binding stoichiometries listed in Table 1) in the positively charged area of the binding pocket, whereas a single Na^+^ ion was added when necessary in the area of the central acidic residue. Bicarbonate ions were oriented with the proton toward the central acidic residue and the carbonate group toward the positively charged area of the binding pocket (Fig. S5). The same starting positions and orientations for the anions (Fig. S5) were used in the protonated hAE1 and hNBCe1 systems. Protonation of the central acidic residue found in the binding pockets of hAE1 and hNBCe1 was done at the first step of the CHARMM-GUI model building protocol (71, 72) for some of the models before the protein was embedded in the POPC bilayer as described above.

#### MD simulations setup

The systems were then equilibrated following the six-step constrained equilibration procedure implemented in CHARMM-GUI (98) and were subjected to at least 250 ns long nonconstrained production MD runs in isothermal-isobaric (NPT) conditions (1 atm and 310.15 K, maintained with Langevin dynamics) in the absence of external potential. All simulations were performed with the Nanoscale Molecular Dynamics (NAMD) 2.12 program (99) employing the CHARMM36 force field for proteins and lipids (83, 100) the available CGEenFF parameters (88, 101) for carbonate and bicarbonate ions and the TIP3P model for water (102). Nonbonded interactions were cut-off and switched off at 12 and 10 Å, and long-range electrostatic interactions were treated by the Particle-Mesh-Ewald algorithm (103).

The analysis of all MD trajectories was done with Visual Molecular Dynamics (VMD) 1.9.3 (104). In-house TCL scripts were used for extraction of contact frequencies from the aligned and centered MD trajectories. The ion density maps were generated with the VolMap tool of VMD.

### ANTON2 MD simulations of apo-hAE1 in NaCl and NaCl+NaHCO_3_ solutions

For assessment of Cl^−^ and HCO_3_^−^ permeation within the hAE1 OF cavity, long MD runs (1.2 μs) were performed with the Anton 2 supercomputer of apo-hAE1 (with either deprotonated or protonated E681) with 150 mM NaCl solution or in a 75 mM NaCl +75 mM NaHCO_3_ mixture (with sizes and lipid compositions as described above) under applied voltage of 0.055 kcal mol^−1^ Å^−1^ e^−1^ (105). The Anton two software version 1.27.0 from D. E. Shaw Research was used for 1.2 μs production runs using the purpose-built Anton 2 supercomputer (105). These simulations were carried out with the same periodic boxes specified above in a NVT ensemble (MD ensemble with constant number of particles (N), volume (V), and temperature (T)) at 310 K to prevent fluctuations in the system size that would alter the applied voltage, because the virial used in the constant pressure algorithm is no longer well defined in an NPT system when applying an external constant electric field. A 2.5 fs time step was used with nonbonded long-range interactions computed every 6 fs using the Reference System Propagation Algorithm, multiple-time-step algorithm (106). The multiintegrator (multigrator) algorithm developed in-house by D. E. Shaw Research (107) was used for temperature and semiisotropic pressure coupling, whereas their *u*-series algorithm was used to handle long range electrostatic interactions (105, 108). Long MD simulations with apo-hNBCe1 were not done because of the lower resolution of the structure and the large number of unresolved residues. Instead, a 250 ns MD simulation of apo-hNBCe1 in 150 mM Na_2_CO_3_ with the setup for 250 ns MD simulations, outlined in the previous section was performed for assessment of the ion dynamics in the cavity of hNBCe1.

#### HEK293 cell culture and transfection

The HEK293 cells were grown at 37 °C in media containing Dulbecco's Modified Eagle Medium with l-glutamine (200 mg l^−1^), 10% fetal bovine serum, and 1% penicillin-streptomycin in a 5% CO_2_ and cultured onto polyethylenimine-coated coverslips for the functional studies and 60-mm dishes for the sulpho-NHS-SS-biotin experiments. The cells were transfected with lipofectamine 2000 (Thermo Fisher Scientific) according to the manufacturer's protocol except that after a 2 h exposure, the transfection solution was removed, and the cells were studied 24 h later. The cells were transfected with various human AE1 and NBCe1-A constructs or an empty pcDNA3.1 vector.

#### Measurement of pH_i_ and H^+^/base flux

Internal pH (pH_i_) was measured 24 h following transient transfection with the various constructs in HEK293 cells grown on polyethylenimine-coated coverslips. The coverslips were placed on the stage of a microscope fluorometer in a custom designed chamber and then loaded at room temperature for ~20 min with the fluorescent pH_i_ probe 2′,7′-bis(2-carboxyethyl)-5(6)-carboxyfluorescein (BCECF) using 2′,7′-bis(2-carboxyethyl)-5(6)-carboxyfluorescein tetrakis(acetoxymethyl) ester (BCECF-AM) (Life Technologies). The dye loading solution contained 140 mM KCl, 5 mM TMACl, and 5 mM Hepes. In the experiments, the fluorescence excitation ratio (500 nm/440 nm; 530 nm emission) was obtained from ~200 cells and averaged. The coverslips were continuously perfused at 2 ml min^−1^ (37 °C) with the bathing solutions. The intracellular fluorescence excitation ratio was calibrated at the end of each experiment using 26 μM nigericin (Sigma-Aldrich) and 5 μM valinomycin (Sigma-Aldrich) to equilibrate pH_i_, external pH, and K^+^. The intrinsic cell buffer capacity (β_*i*_) was measured over a range of intracellular H^+^ (H_in_^+^) values in Hepes-buffered solutions using NH_4_^+^ addition/removal protocols and calculated as Δ[NH_4_^+^]/Δ[H_in_^+^]. The bicarbonate buffer capacity β_*HCO3*_ was calculated as 2.3 × [HCO_3_^−^]_in_. The total buffer capacity was therefore β_*i*_ + β_*HCO3*_. The rate of change of pH_i_ (dpH_i_/d*t*) was measured in the initial 10 to 15 s after a bath solution switch and converted to the rate of change of [H_in_^+^] (d[H_in_^+^]/d*t*), and the H^+^ flux (mM s^−1^) was calculated as (β_*i*_ + β_*HCO3*_) × (d[H_in_^+^]/d*t*).

#### Anion exchange (AE1) and Na-driven base (NBCe1-A) transport assays

(1) Anion exchange transport assay: The cells were initially bathed in the following Na^+^-free Cl^−^-free solution: 115 mM tetramethylammonium hydroxide, 115 mM gluconic acid lactone, 2.5 mM K_2_HPO_4_, 7.5 mM calcium gluconate, 1 mM magnesium gluconate, 24 mM tetramethylammonium bicarbonate, 5% CO_2_, pH 7.4, and 30 μM 5-(N-ethyl-N-isopropyl)-amiloride (EIPA). AE1-mediated Cl^−^ flux was induced by switching to the following Na^+^-free Cl^−^-containing solution: 115 mM tetramethylammonium chloride, 2.5 mM K_2_HPO_4_, 1 mM CaCl_2_, 1 mM MgCl_2_, 24 mM tetramethylammonium bicarbonate, 5% CO_2_, pH 7.4, and 30 μM EIPA. (2) Na^+^-driven base transport assay: The cells were initially bathed in the following Na^+^-free, Cl^−^-containing, bicarbonate-free solution: 140 mM tetramethylammonium chloride, 2.5 mM K_2_HPO_4_, 1 mM CaCl_2_, 1 mM MgCl_2_, 5 mM Hepes, pH 7.4, and 30 μM EIPA. NBCe1-A–mediated Na^+^-driven base flux was induced by switching to the following Na^+^- and Cl^−^-containing solution: 115 mM NaCl, 2.5 mM K_2_HPO_4_, 1 mM CaCl_2_, 1 mM MgCl_2_, 24 mM NaHCO_3_, 5% CO_2_, pH 7.4, and 30 μM EIPA. The statistical analysis of the flux measurements is presented in Tables S1 and S2.

#### Sulfo-NHS-SS-biotin plasma membrane labeling

Sulfo-NHS-SS-biotin was used to label plasma membrane proteins. Streptavidin-agarose resin was used to pull down these proteins. The following protocol was utilized; the cells were washed with phosphate buffered saline at room temperature, pH 8.0, 24 h following transfection and were then incubated at 4 °C for 30 min (pH 8.0) with 1.1 mM sulfo-NHS-SS-biotin (Thermo Fisher Scientific). 50 mM Tris buffer at 4 °C (140 mM NaCl, pH 8.0) was used to stop the reaction. The cells were collected and washed with phosphate buffered saline, then lysed on ice in a solution containing 150 mM NaCl, 0.5% sodium deoxycholate (Thermo Fisher Scientific), 1% (vol/vol) octylphenoxy poly(ethyleneoxy)ethanol (Igepal) (Sigma-Aldrich), 10 mM Tris HCl, 5 mM EDTA (Sigma-Aldrich), pH 7.5, with protease inhibitors (Roche Life Sciences). The insoluble material was pelleted over 10-min centrifugation at 20,000*g* (4 °C), and then, the supernatant (containing >90% of the plasma membrane protein fraction) was collected and then incubated on a rotating shaker at 4 °C for 4 h with streptavidin-agarose resin (50 μl) (Thermo Fisher Scientific). The resin was pelleted by brief centrifugation and washed with the lysis buffer at 60 °C for 5 min with 2 × SDS buffer containing 2% 2-mercaptoethanol (EMD Millipore, Billerica, MA) to elute bound proteins. For the lysate detection, the cells were lysed in lysis buffer (150 mM NaCl, 0.5% sodium deoxycholate [Thermo Fisher Scientific], 1% (vol/vol) Igepal [Sigma-Aldrich], 10 mM Tris HCl, 5 mM EDTA [Sigma-Aldrich] pH 7.5), and AE1 was pulled down using the AE1 2-M anti-human monoclonal antibody (Alpha Diagnostics) (1:1000 dilution), and NBCe1-A was pulled down with the previously described rabbit polyclonal anti-human NBCe1-A antibody (1:1000 dilution).

#### SDS-PAGE and immunoblotting

The protein samples were initially resolved on 7.5% polyacrylamide gels and then transferred to polyvinylidene difluoride membranes. The expression levels of the pulled down biotinylated proteins and whole cell lysates were assessed by probing the blots with either the mouse monoclonal AE1 2-M antibody (1:10,000 dilution) or the rabbit polyclonal NBCe1-A antibody (1:10,000 dilution) in Tris buffered saline with polyethylene glycol sorbitan monolaurate (TBSTM) buffer (0.1% (vol/vol) Tween 20; 137 mM NaCl, 20 mM Tris, pH 7.5, containing 5% (wt/vol) nonfat milk). After 1 h incubation at room temperature, the blots were washed with TBST and then probed with Peroxidase AffiniPure Donkey Anti-Mouse IgG (H + L) (Jackson ImmunoResearch Laboratories, Inc) at 1:10,000 dilution or Peroxidase AffiniPure Mouse Anti-Rabbit IgG (H + L) (Jackson ImmunoResearch Laboratories, Inc) at 1:10,000 dilution in TBSTM buffer and incubated at room temperature for 1 h. The blots were washed with TBST, and signals were detected with Enhanced Chemiluminescent (ECL) Western Blotting Detection Reagent (GE HealthCare).

## Data availability

All necessary data is provided in the manuscript. The custom scripts used for analysis of the MD trajectories will be provided upon request by the corresponding author Sergei Noskov.

## Supporting information

This article contains supporting information.

## Conflict of interest

The authors declare that they have no conflicts of interest with the contents of this article.
