# Supplementary material for: Identification of multiple substrate binding sites in SLC4 transporters in the outward-facing conformation: Insights into the transport mechanism
Source: J Biol Chem. 2021 Apr 28;296:100724. doi: 10.1016/j.jbc.2021.100724 (PMC8191340; doi:10.1016/j.jbc.2021.100724)
Supplement: Figures S1 to S15 and Tables S1 and S2 [file mmc1.pdf]

Supporting Information for

Identification of multiple substrate binding sites in SLC4 transporters in the outward-facing conformation: insights into the transport mechanism

Hristina R. Zhekova[a], Alexander Pushkin[b], Gülru Kayık[a], Liyo Kao[b], Rustam Azimov[b], Natalia Abuladze[b], Debra Kurtz[b], Mirna Damergi[a], Sergei Noskov[a,*] and Ira Kurtz[b,c,*]

[a]Centre for Molecular Simulation, Department of Biological Sciences, University of Calgary, 2500 University Dr. NW, Calgary, Alberta T2N 1N4, Canada; [b]Department of Medicine, Division of Nephrology, David Geffen School of Medicine, University of California, Los Angeles, CA 90095, USA; [c]Brain Research Institute, University of California, Los Angeles, CA 90095, USA

Corresponding authors:
Ira Kurtz, Professor, Department of Medicine, Division of Nephrology, David Geffen School of Medicine, University of California, Los Angeles, California and Brain Research Institute, University of California, Los Angeles, California, CA 90095
e-mail: ikurtz@mednet.ucla.edu

or

Sergei Noskov, Professor, Centre for Molecular Simulation, Department of Biological Sciences, University of Calgary, 2500 University Dr. NW, Calgary, Alberta T2N 1N4, Canada
e-mail: snoskov@ucalgary.ca

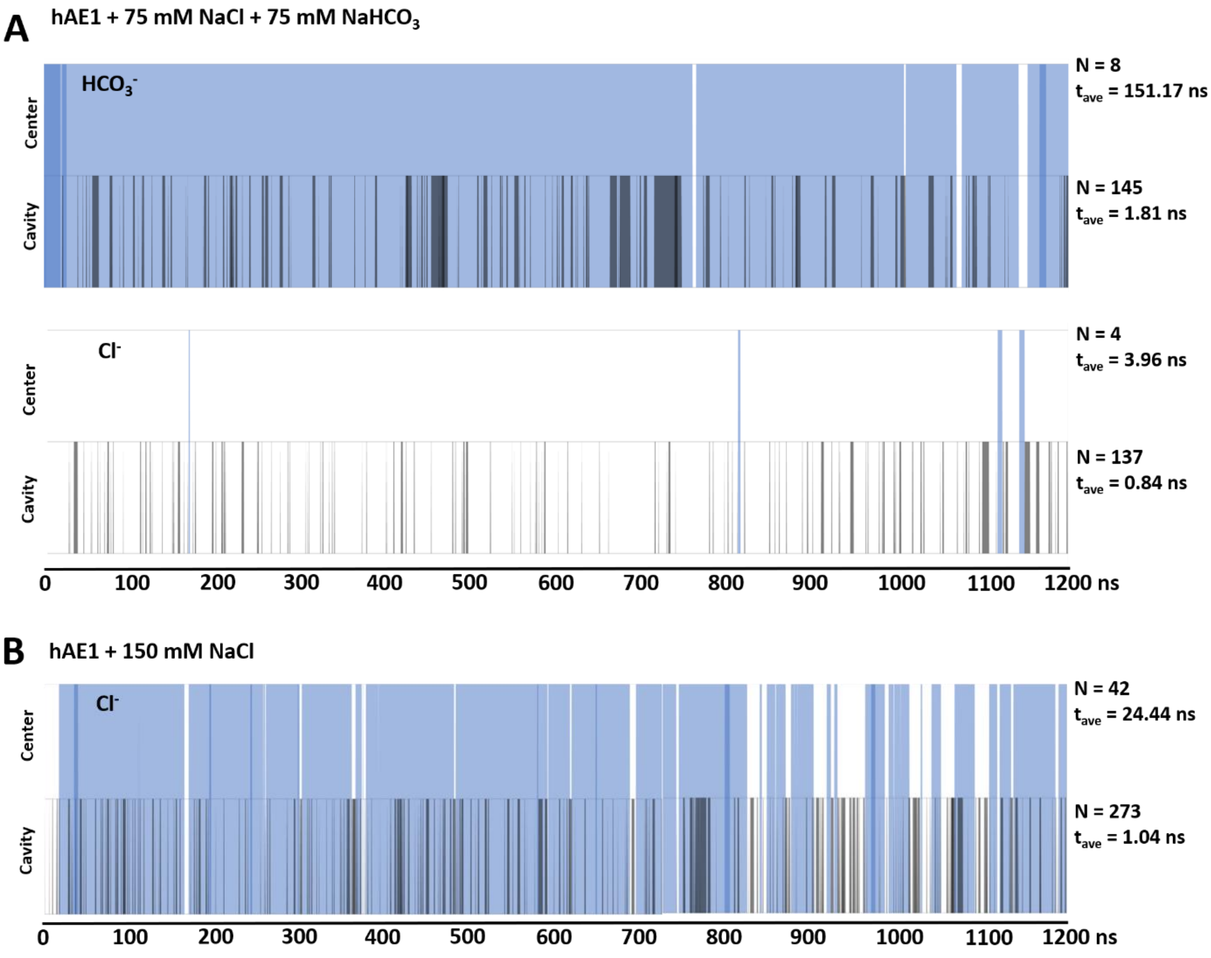

Fig. S1 Occupation by HCO_3^- and Cl^- ions of the cavity (grey bars) and central areas (blue bars) of hAE1 (defined in Fig. S2) during the 1.2 μs MD simulations in apo-hAE1 in A) equimolar solution of 75 mM NaCl + 75 mM $NaHCO_3$ and B) apo-hAE1 in 150 mM NaCl solution. The span of the colored areas reflects the amount of time during which HCO_3^- or Cl^- ions are present in the permeation cavity or protein center. The overlap of bars (areas in deeper grey and blue color) indicates the simultaneous presence of more than one anion in the cavity and/or protein center (in such cases, often one anion is coordinated in the protein center area, while the other is in the upper portion of the cavity, see Fig. S2). The number of unique anion entry events (N) and the average residence times (t_{ave}) in the permeation cavity and protein center are included in the figure.

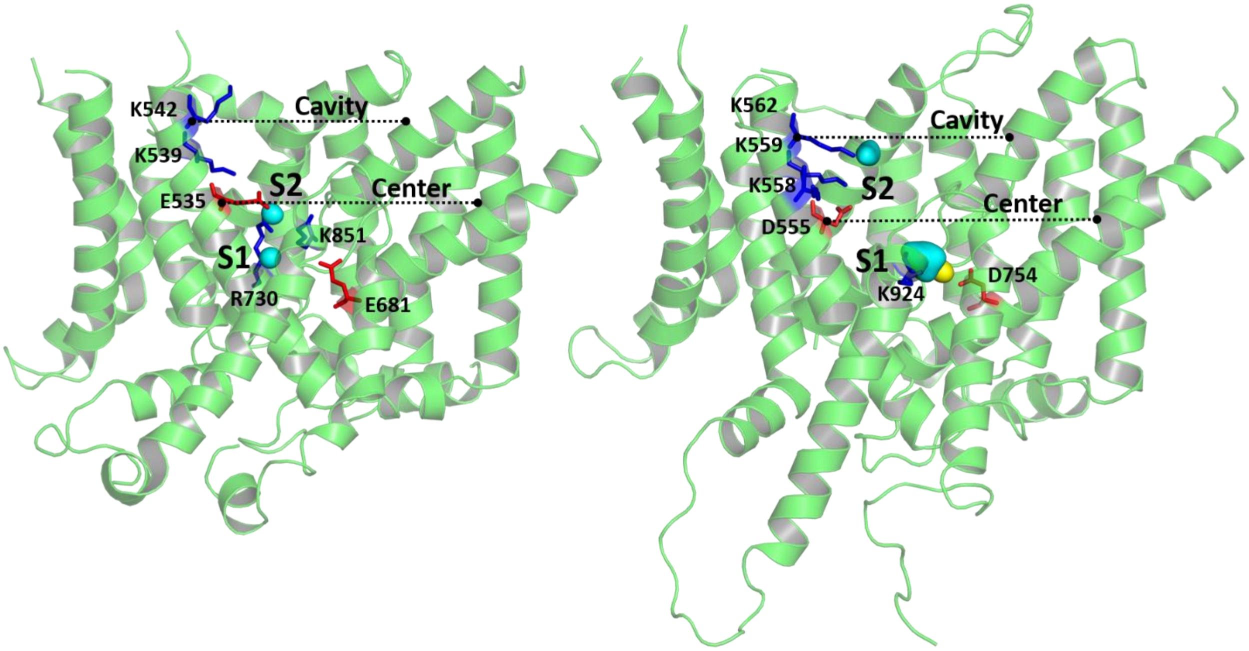

Fig. S2 Definition of cavity and central areas of hAE1 and hNBCe1 used in the manuscript. The protein structures with ion densities outlining the location of the putative ion binding sites are taken from Fig. S6. The permeation cavity is defined as the area of the protein below the C_α atom of residue K542 in hAE1 or residue K562 in hNBCe1. The central region is a smaller part of the overall permeation cavity found below the C_α atom of residue E535 in hAE1 or residue D555 in hNBCe1. The central binding site S1 is at the bottom of the central region, while the entry binding site S2 falls in the general area of the cavity (in hNBCe1) or at the border of the central region (in hAE1). The flanking charged residues (red color for acidic and blue color for basic) which are part of sites S1 and S2 are shown as well.

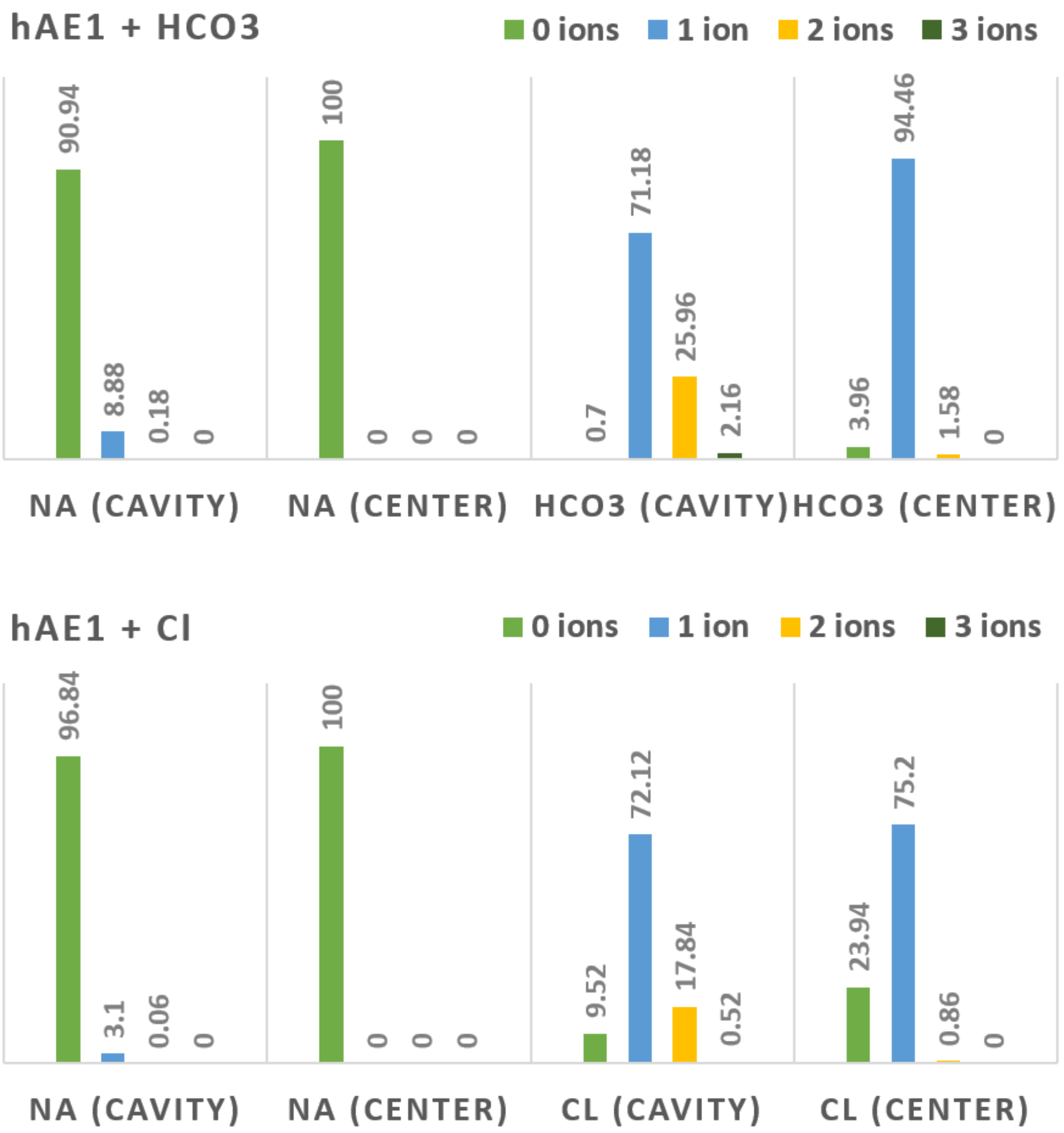

Fig. S3 Number of Na^+, Cl^-, and HCO_3^- ions, which can be found in the cavity and center areas of hAE1 evaluated from 1.2 μs MD simulations of hAE1 in equimolar 75 mM $NaHCO_3$ + 75 mM NaCl solution and hAE1 in 150 mM NaCl solution. The results are presented as % of MD trajectory steps, in which one can find N ions (N varies from 0 to 3) of a single type in the permeation cavity of hAE1. The cavity and center definitions are outlined in Fig. S2.

hAE1 + Cl^- (Trial 2)

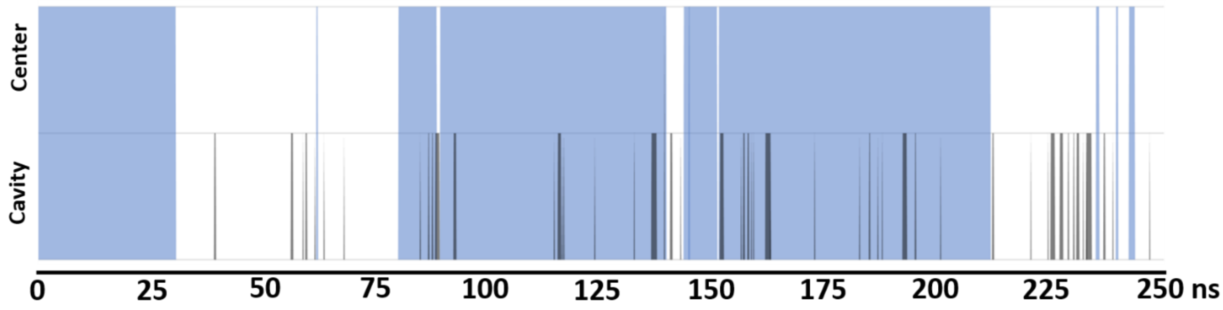

hAE1 + Na^+ + Cl^-

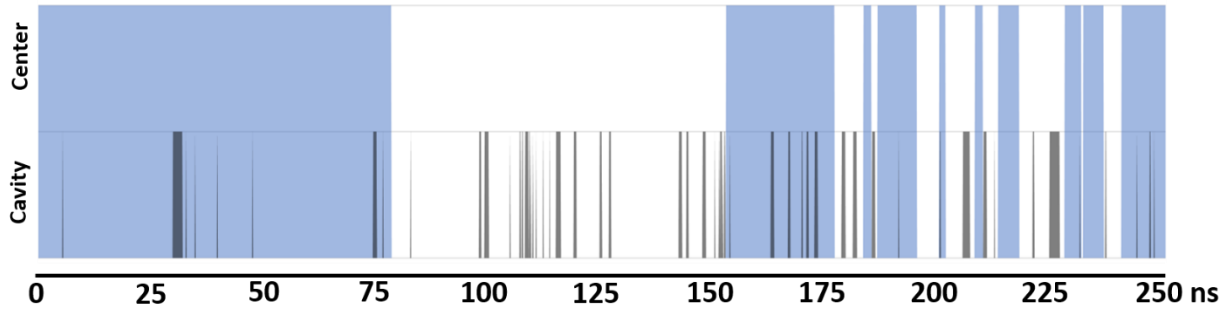

hAE1 + protE681 + Cl^-

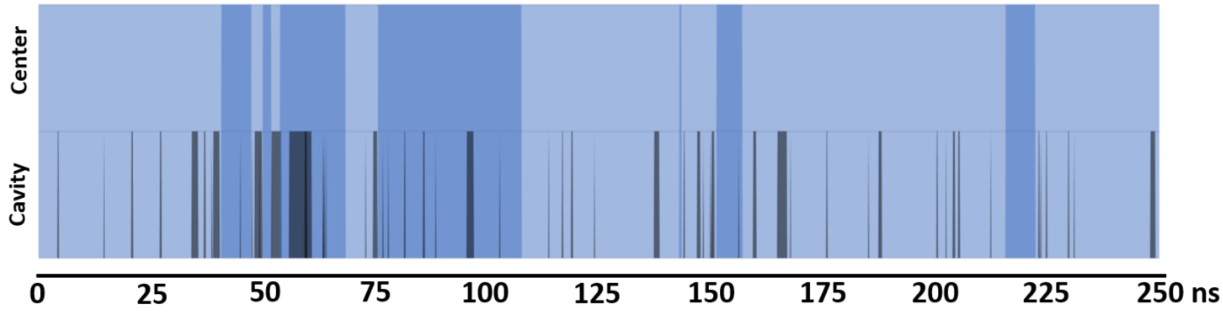

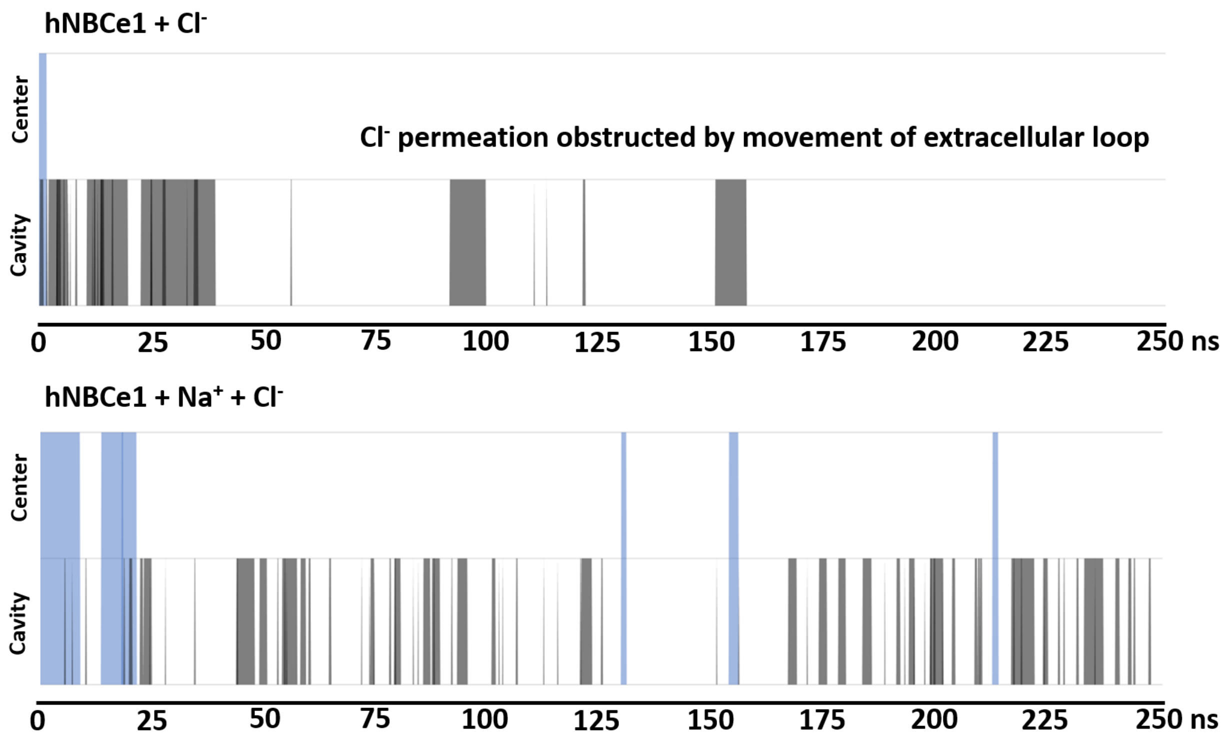

Fig. S4 Dynamics of the Cl⁻ ions within the cavity (grey bars) and central areas (blue bars) of hAE1 and hNBCe1 (defined in Fig. S2) in the systems of Table 1, marked with the * symbol, where the initially bound Cl⁻ dissociated from site S1 and was replaced by another Cl⁻ from the surrounding solution. The span of the colored areas reflects the amount of time during which Cl⁻ ions are present in the permeation cavity or protein center. The overlap of bars (areas in deeper grey and blue color) indicates the simultaneous presence of more than one Cl⁻ in the cavity and/or protein center. This overlap is especially pronounced in the hAE1 system protonated at E681. The ion permeation of the hNBCe1 + Cl⁻ system was obstructed after 75 ns due to repositioning of a large extracellular loop above the cavity.

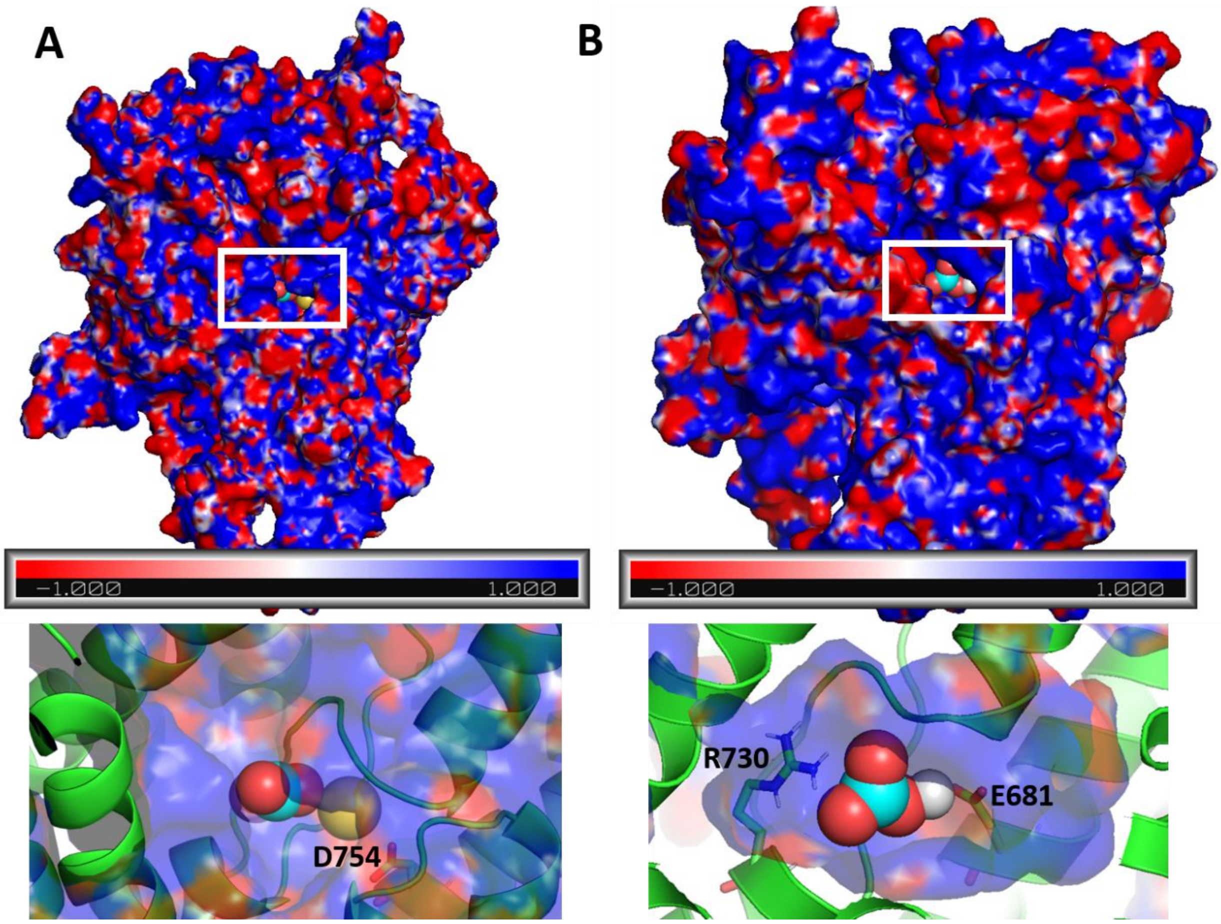

Fig. S5 Poisson-Boltzmann maps (kcal/mol) of A) hNBCe1 and B) hAE1 viewed from above, perpendicular to the membrane. The ion binding area in the full protein frame is marked with a white box. Colors: red - negatively charged regions attractive to positively charged ions; blue – positively charged areas attractive to negatively charged ions. The initial positions of the bound Na^{+}+CO_3^{2-} (in hNBCe1) and HCO_3^{-} (in hAE1) in sphere representation are shown (below) in site S1, oriented according to the protein charges, arising from nearby arginine, aspartate, and glutamate residues (shown as sticks).

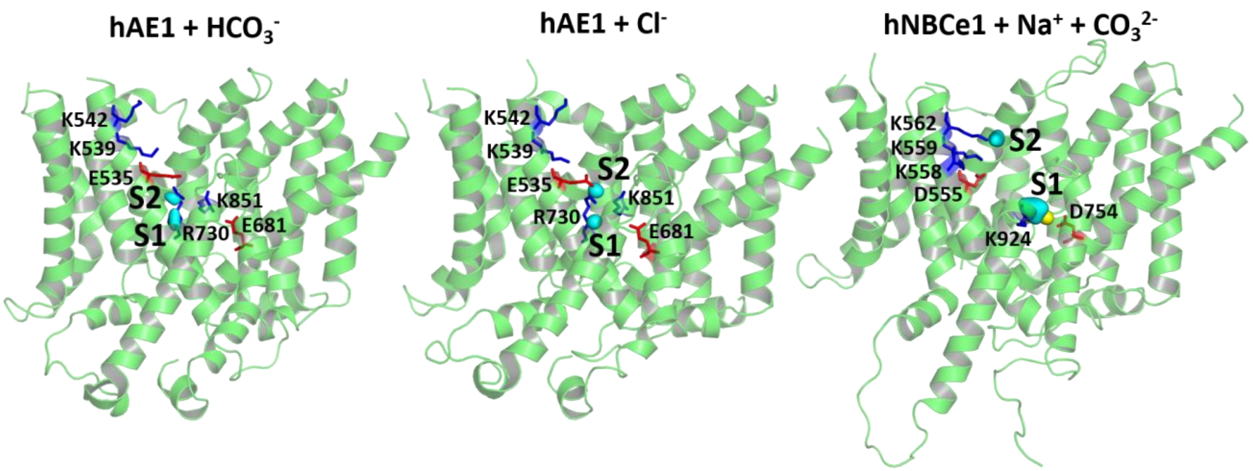

Fig. S6 Location of two putative anion binding sites S1 (central binding site) and S2 (entry binding site) identified from 250 ns MD simulations of hAE1 loaded with HCO_3^-, hAE1 loaded with Cl^-, and hNBCe1, loaded with Na^+ and CO_3^{2-} in site S1. The anion density maps in hAE1 and hNBCe1 are colored in cyan while the Na^+ density map in hNBCe1 is colored in yellow. The CO_3^{2-} density in site S2 of hNBCe1 is plotted from the MD simulation of hNBCe1+ CO_3^{2-} where the CO_3^{2-} ion migrated toward site S2 due to the absence of a stabilizing Na^+ at the beginning of the MD simulation.

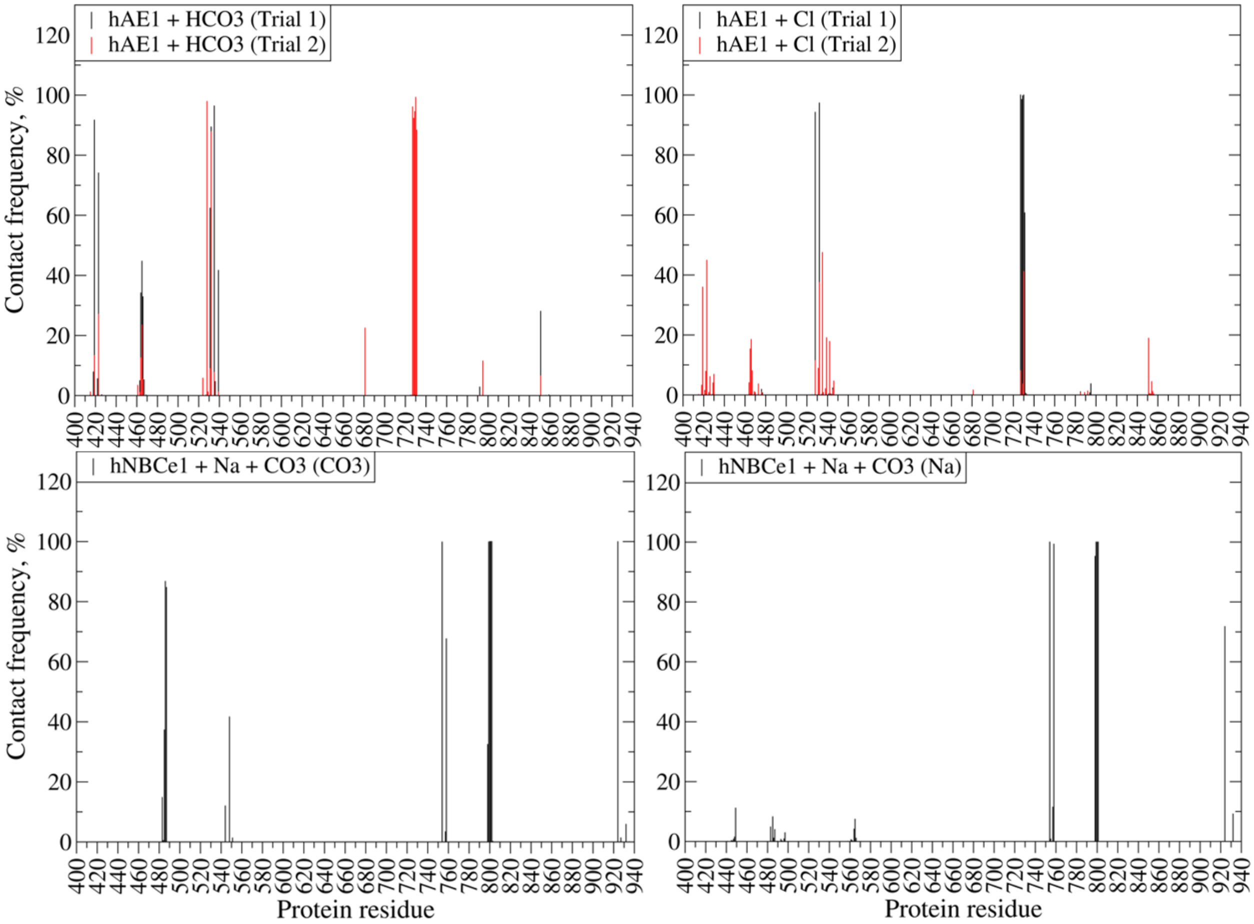

Fig. S7 Contact frequencies of the HCO_3^-, Cl^-, CO_3^{2-}, and Na^+ calculated from two independent 250 ns MD simulations of the unprotonated wild type hAE1 bound to HCO_3^- or Cl^- and a single 250 ns MD simulation of the unprotonated wild type hNBCe1 bound to one Na^+ and one CO_3^{2-} (Table 1).

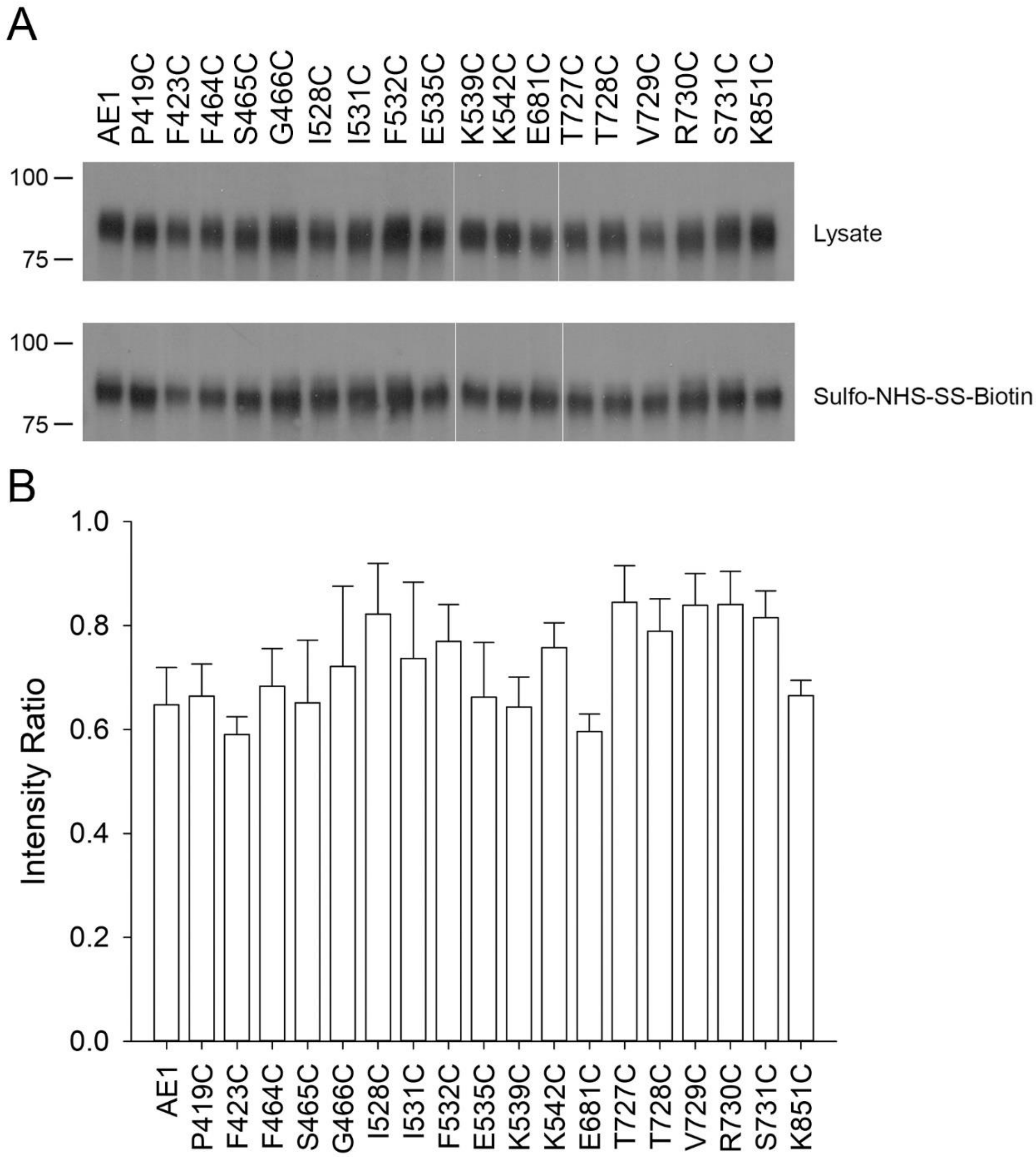

Fig. S8 Membrane expression of the studied hAE1 mutants. A) Representative experiment showing immunoblot analysis of cell lysate and cell surface expression of wild-type and mutant hAE1 proteins. The positions of molecular weight markers (kDa) are shown on the left. Blot splicing is indicated with vertical white lines. B) Densitometry analysis of the ratio of cell-surface to lysate hAE1 protein expression. One-way ANOVA and Dunnett's test were used to compare multiple study group means with WT hAE1. Mutant hAE1 data was not statistically different from WT hAE1. Results are depicted as mean ± SEM (n=3 separate experiments).

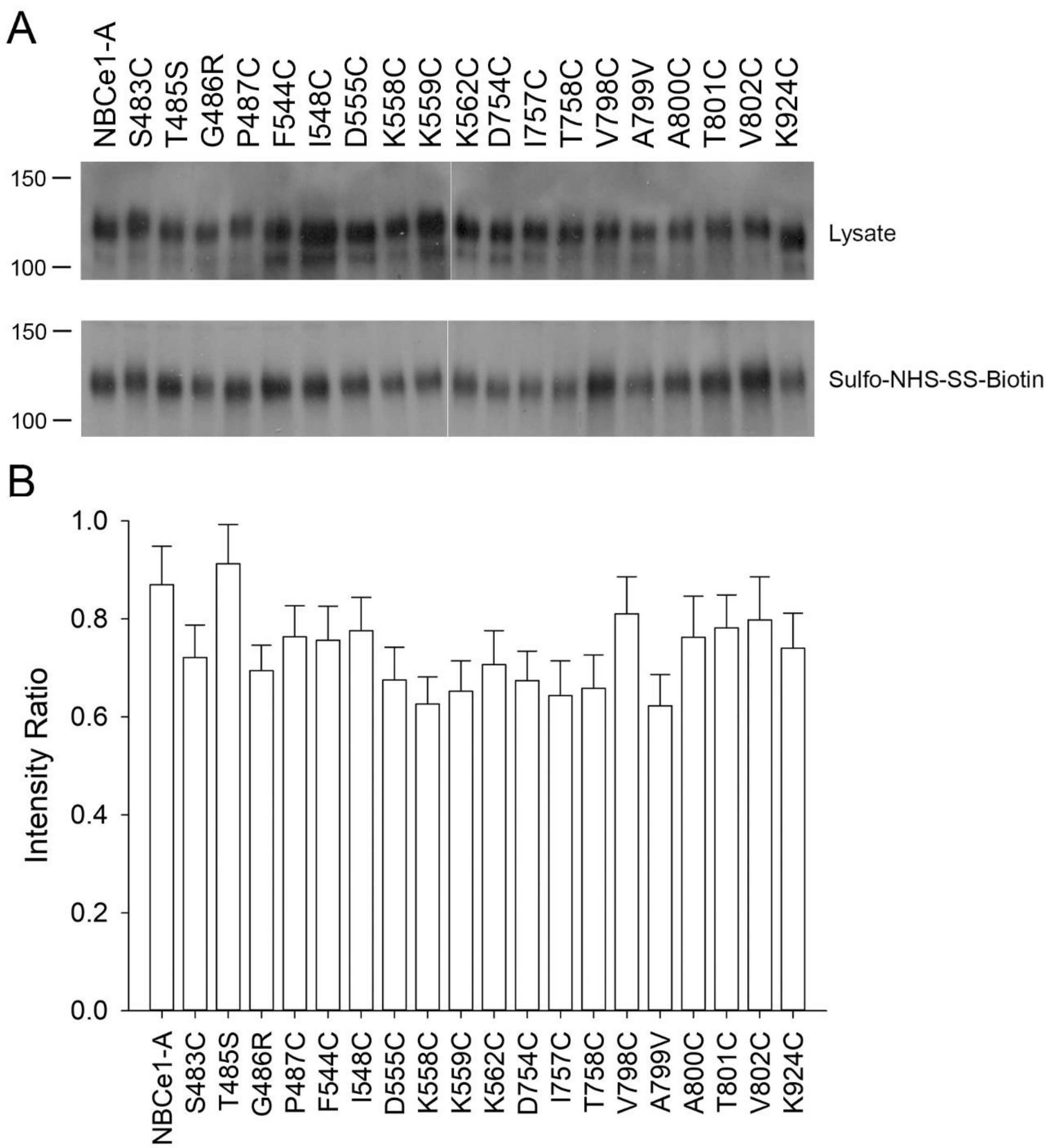

Fig. S9 Membrane expression of the studied hNBCe1 mutants. A) Representative experiment showing immunoblot analysis of cell lysate and cell surface expression of wild-type and mutant hNBCe1-A proteins. The positions of molecular weight markers (kDa) are shown on the left. Blot splicing is indicated with a vertical white line. B) Densitometry analysis of the ratio of cell-surface to lysate hNBCe1-A protein expression. One-way ANOVA and Dunnett's test were used to compare multiple study group means with WT hNBCe1-A. Mutant hNBCe1-A data was not statistically different from WT hNBCe1-A. Results are depicted as mean ± SEM (n=3 separate experiments).

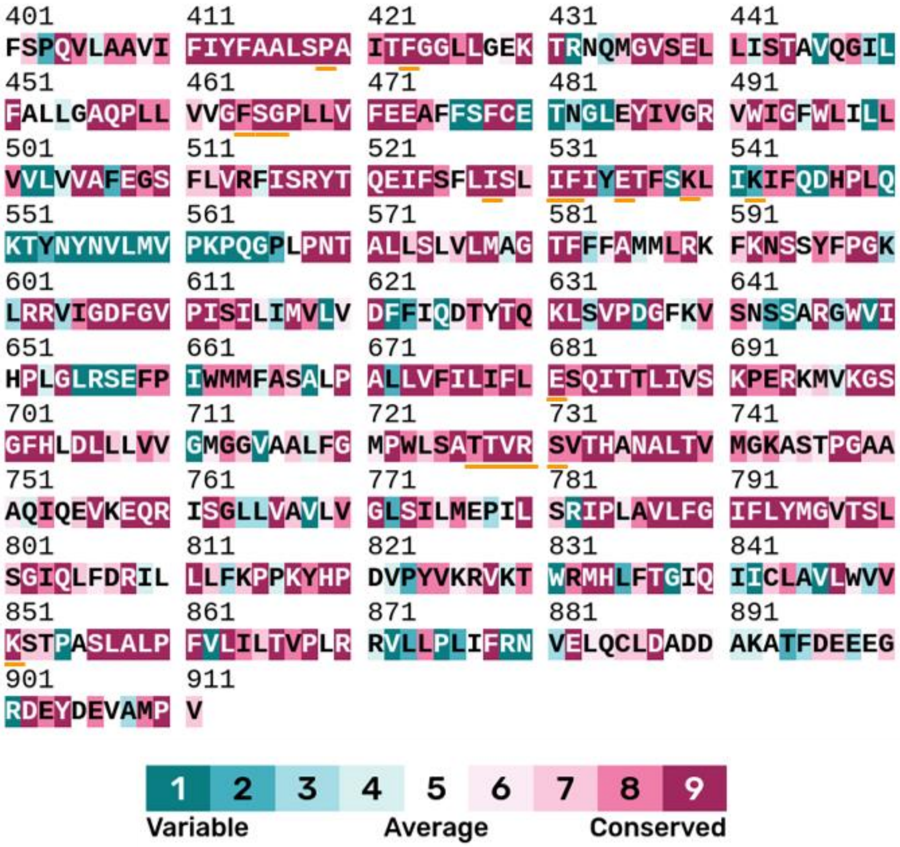

Fig. S10 ConSurf-DB scores of hAE1. The residues of sites S1 and S2 are underlined in orange. (https://consurfdb.tau.ac.il/main_output.php?pdb_ID=4YZF&view_chain=A&unique_chain=4YZFA)

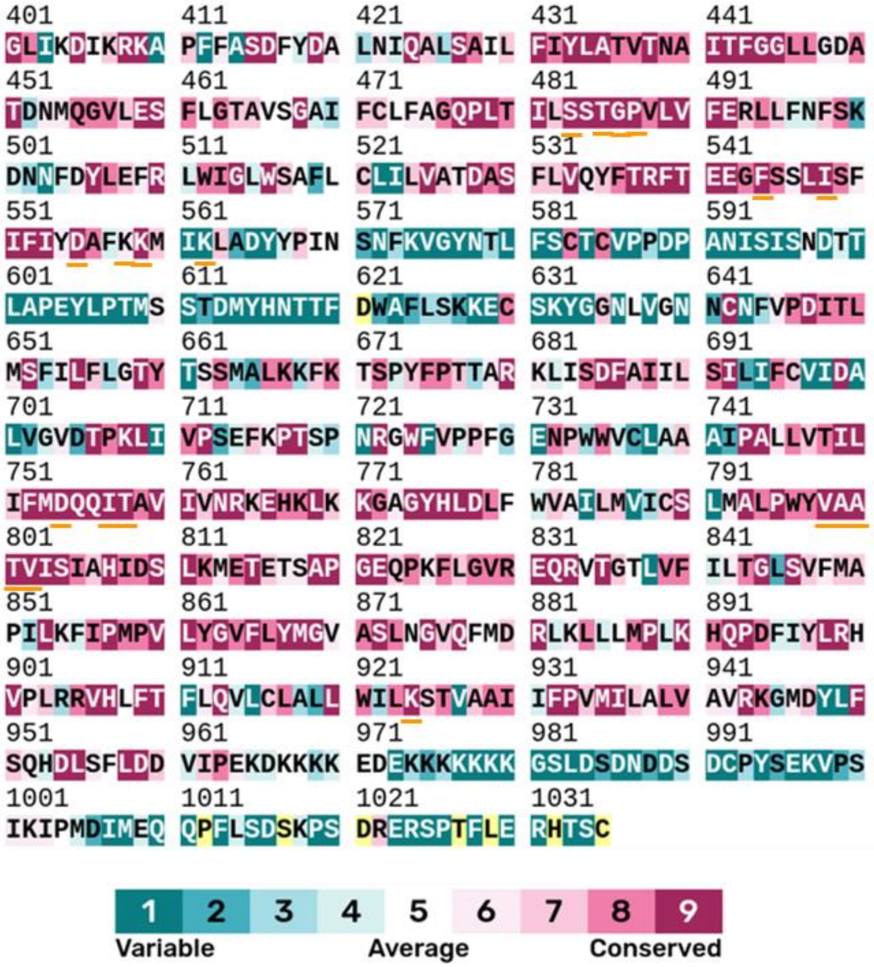

Fig. S11 ConSurf-DB scores of hNBCe1. The residues of sites S1 and S2 are underlined in orange. (https://consurfdb.tau.ac.il/main_output.php?pdb_ID=6CAA&view_chain=A&unique_chain=6CAAA)

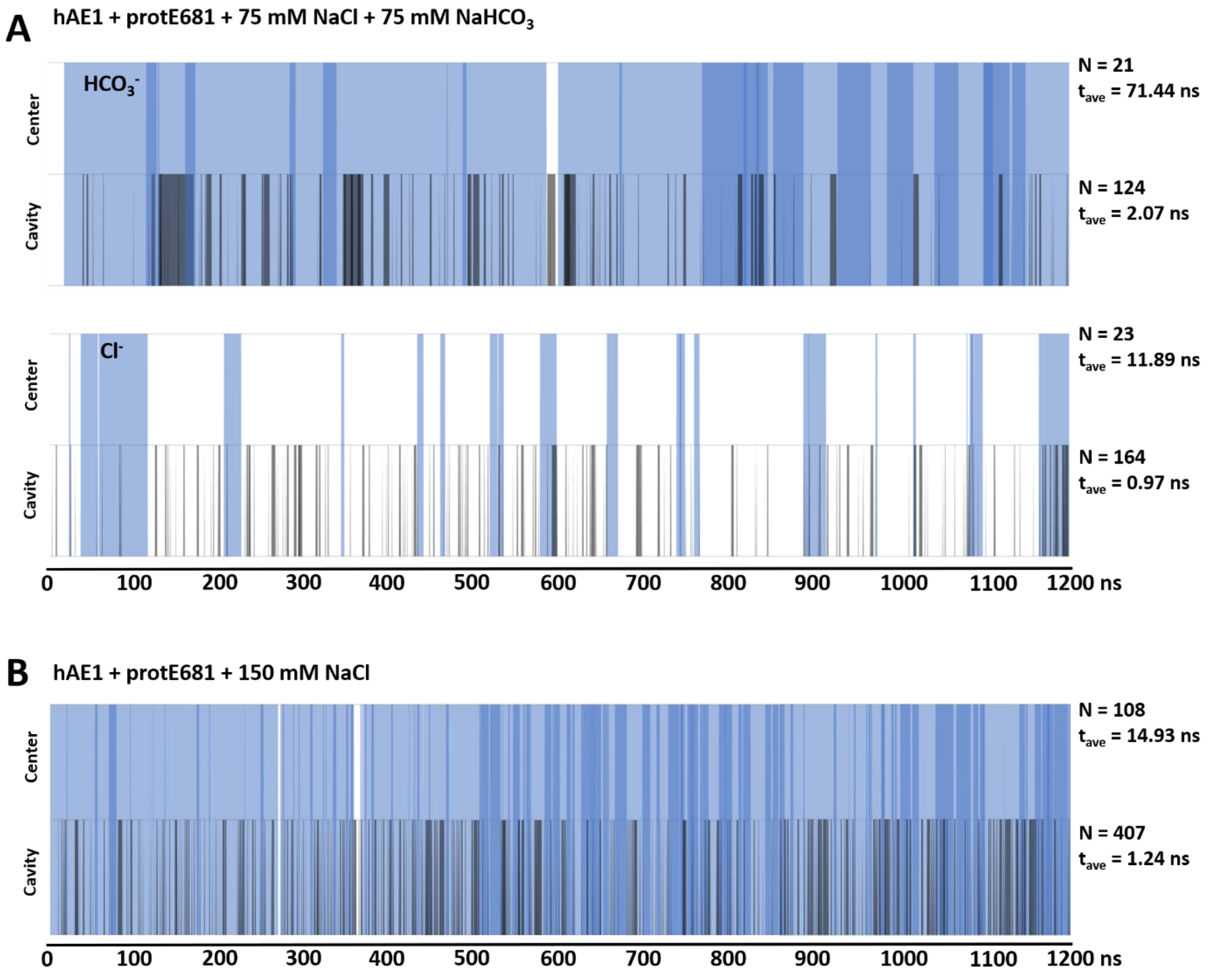

Fig. S12 Occupation by HCO_3^- and Cl^- ions of the cavity (grey bars) and central areas (blue bars) of hAE1 (defined in Fig. S2) during the 1.2 µs MD simulations in apo-hAE1, protonated at E681, in A) equimolar solution of 75 mM NaCl + 75 mM $NaHCO_3$ and B) apo-hAE1 in 150 mM NaCl solution. The span of the colored areas reflects the amount of time during which HCO_3^- or Cl^- ions are present in the permeation cavity or protein center. The overlap of bars (areas in deeper grey and blue color) indicates the simultaneous presence of more than one anion in the cavity and/or protein center. The number of unique anion entry events (N) and the average residence times (t_{ave}) in the permeation cavity and protein center are included in the figure.

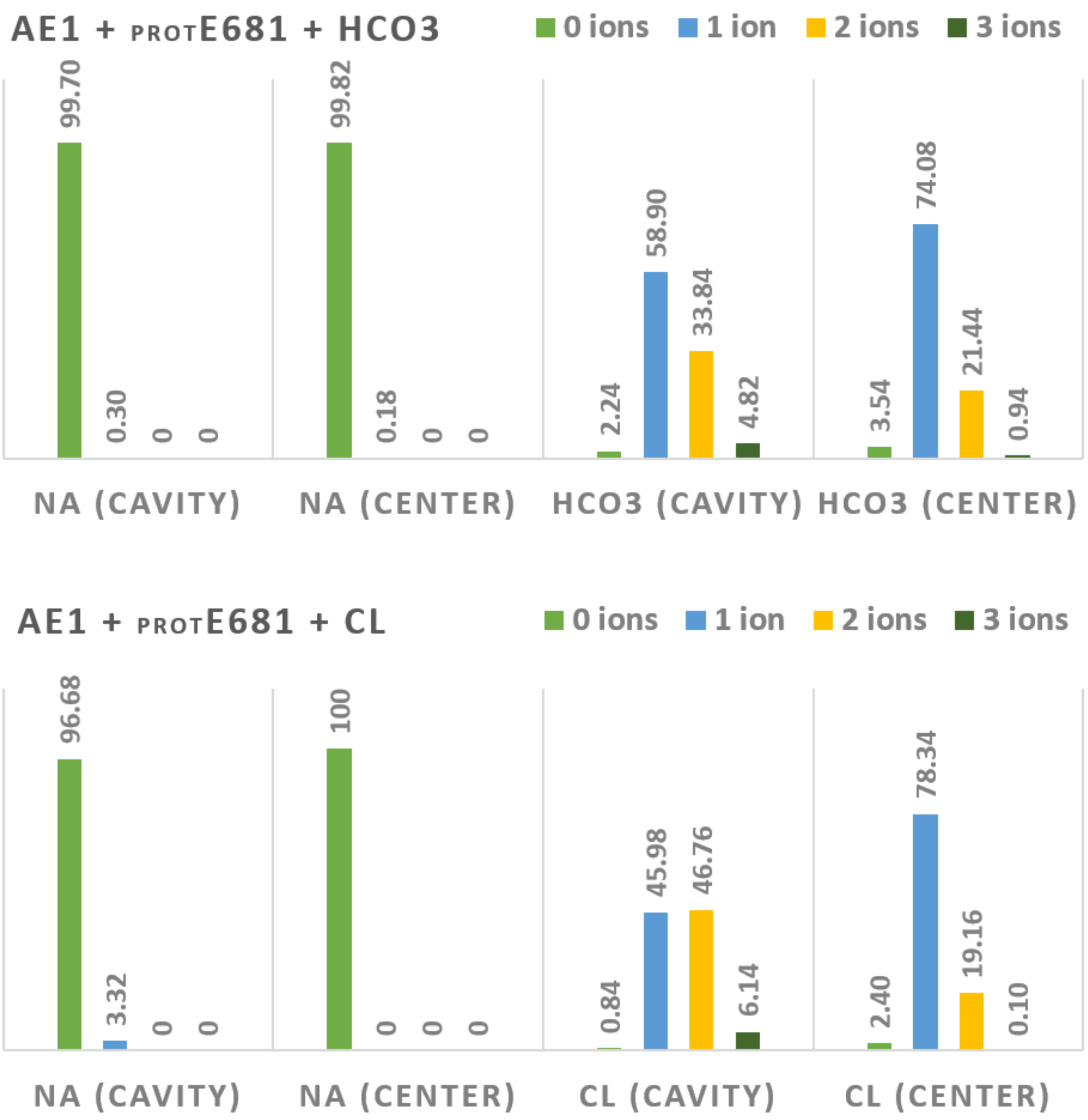

Fig. S13 Number of Na^+, Cl^-, and HCO_3^- ions, which can be found in the cavity and center areas of hAE1 evaluated from 1.2 μs MD simulations of hAE1, protonated at E681, in equimolar 75 mM $NaHCO_3$ + 75 mM NaCl solution and hAE1 in 150 mM NaCl solution. The results are presented as % of MD trajectory steps, in which one can find N ions (N varies from 0 to 3) of a single type in the permeation cavity of hAE1. The cavity and center definitions are outlined in Fig. S2.

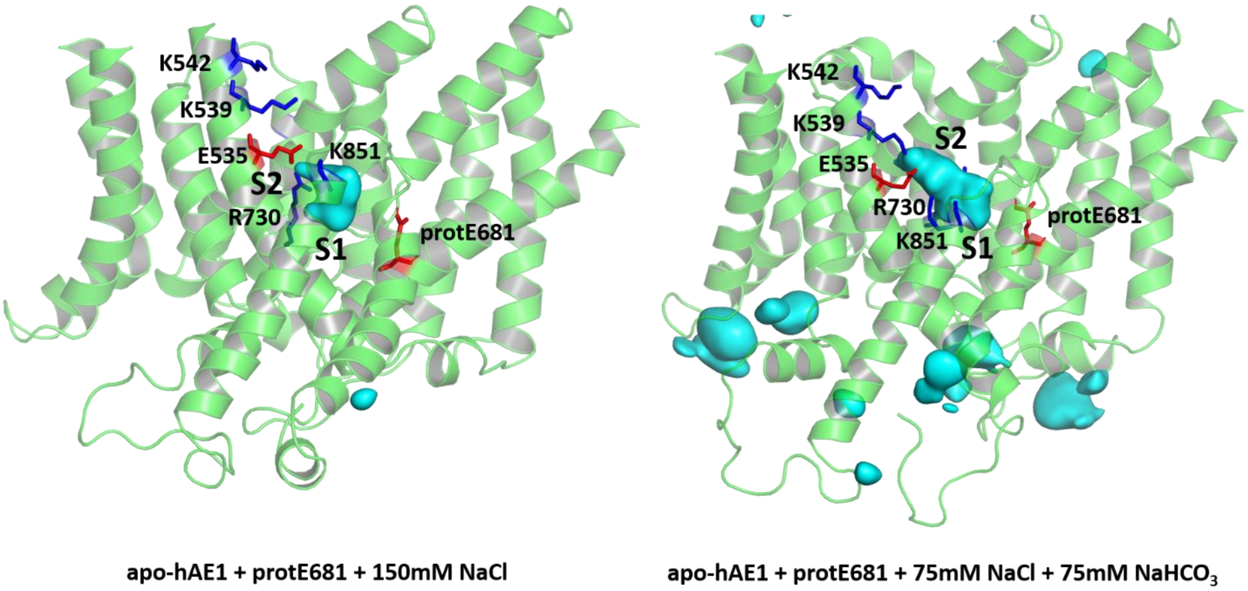

Fig. S14 Anion density maps (isovalue 0.1), computed from 1.2 μs MD trajectories of apo-hAE1, protonated at E681, in 150 mM NaCl solution (Cl^- density map) or in equimolar 75 mM NaCl + 75 mM $NaHCO_3$ mixture (HCO_3^- density map). The maps demonstrate enhanced anion presence in both putative binding sites (S1 and S2) in the OF permeation cavity of hAE1 due to protonation of E681.

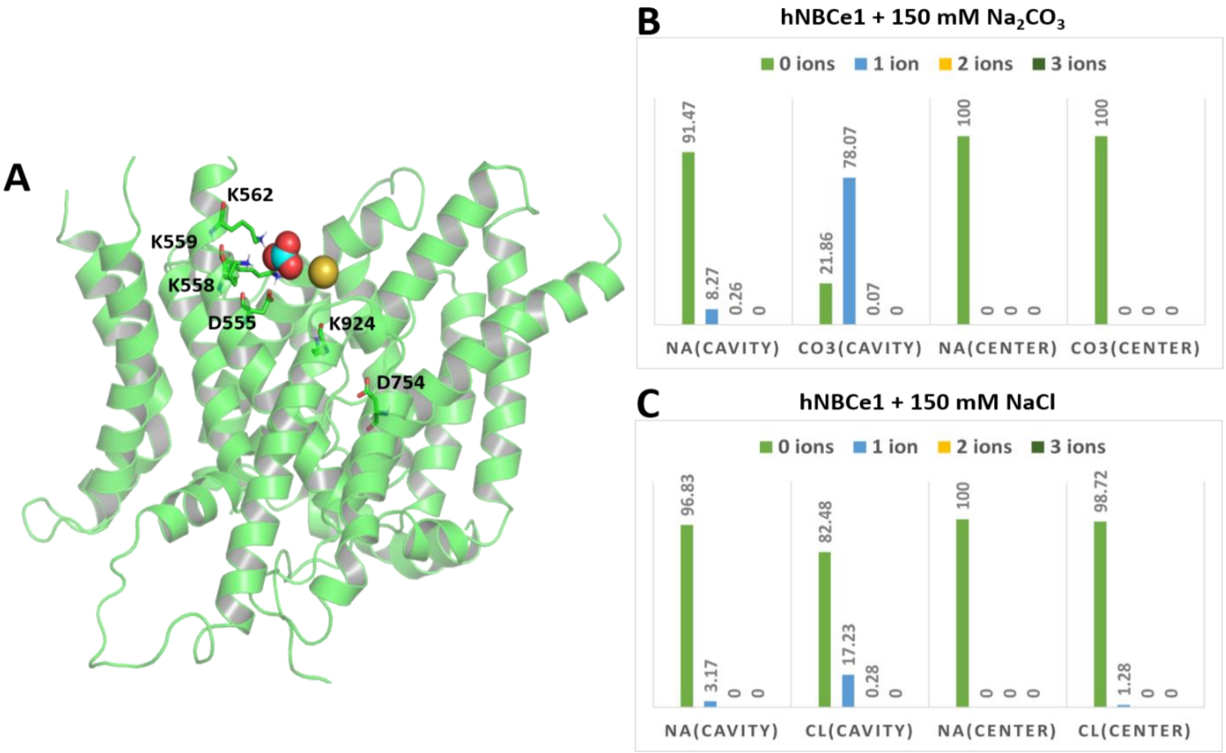

Fig. S15 Ion dynamics in hNBCe1. A) Coordination of CO_3^{2-} by the Lys residues in site S2 which occurs in the majority of MD steps from our 250 ns MD simulation of apo-hNBCe1 in 150 mM Na_2CO_3 solution. In the selected snapshot, a Na^+ ion has been drawn by CO_3^{2-} from the surrounding solution to the protein cavity. B) Number of Na^+ and CO_3^{2-} ions which can be found in the cavity and center areas of hNBCe1 evaluated from 250 ns MD simulations of apo-hAE1 in 150 mM Na_2CO_3 solution. C) Number of Na^+ and Cl^- ions which can be found in the cavity and center areas of hNBCe1 evaluated from 250 ns MD simulations of hNBCe1+Na^++Cl^- in 150 mM NaCl solution (Table 1, the first 25 ns of the trajectory during which the initial Na^+ and Cl^- ions were still in the permeation cavity were discarded for the purpose of this analysis). The results are presented as % of MD trajectory steps in which one can find N ions (N varies from 0 to 3) of a single type in the permeation cavity of hNBCe1. The cavity and center definitions are outlined in Fig. S2.

Table S1 Cl^--driven base flux results in the studied hAE1 constructs.

AE1 Mutation	+ Cl driven flux (mM·sec^{-1})	SEM	p value	n
Mock	-0.055	0.015	p < 0.001	4
WT AE1	-0.583	0.024	–	6
P419C	-0.226	0.015	p < 0.001	18
F423C	-0.170	0.023	p < 0.001	7
F464C	-0.554	0.040	NS	4
S465C	-0.238	0.066	p < 0.001	6
G466C	-0.282	0.040	p < 0.001	8
I528C	-0.358	0.031	p < 0.001	4
I531C	-0.339	0.033	p < 0.001	4
F532C	-0.429	0.019	p < 0.005	12
E535C	-0.223	0.017	p < 0.001	13
K539C	-0.314	0.026	p < 0.001	7
K542C	-0.426	0.045	p < 0.005	7
E681C	-0.347	0.017	p < 0.001	5
T727C	-0.432	0.042	p < 0.001	13
T728C	-0.281	0.021	p < 0.001	14
V729C	-0.264	0.018	p < 0.001	9
R730C	-0.192	0.035	p < 0.001	4
S731C	-0.263	0.022	p < 0.001	5
K851C	-0.325	0.032	p < 0.001	8

Table S2 Na^+-driven base flux results in the studied hNBCe1 constructs.

NBCe1-A Mutation	+Na driven flux ($mM \cdot sec^{-1}$)	SEM	p value	n
Mock	0.107	0.011	$p < 0.001$	8
WT NBCe1-A	0.547	0.020	–	8
S483C	0.178	0.016	$p < 0.001$	7
T485S	0.245	0.019	$p < 0.001$	4
G486R	0.137	0.008	$p < 0.001$	3
P487C	0.321	0.019	$p < 0.001$	4
F544C	0.182	0.022	$p < 0.001$	4
I548C	0.356	0.020	$p < 0.001$	4
D555C	0.321	0.024	$p < 0.001$	6
K558C	0.369	0.032	$p < 0.001$	3
K559C	0.506	0.026	NS	4
K562C	0.503	0.038	NS	6
D754C	0.305	0.022	$p < 0.001$	4
I757C	0.283	0.013	$p < 0.001$	3
T758C	0.265	0.031	$p < 0.001$	4
V798C	0.601	0.055	NS	3
A799V	0.309	0.029	$p < 0.001$	4
A800C	0.513	0.029	NS	7
T801C	0.509	0.020	NS	7
V802C	0.512	0.012	NS	3
K924C	0.213	0.009	$p < 0.001$	3
